# A systematic review of Machine Learning and Deep Learning approaches in Mexico: challenges and opportunities

**DOI:** 10.3389/frai.2024.1479855

**Published:** 2025-01-07

**Authors:** José Luis Uc Castillo, Ana Elizabeth Marín Celestino, Diego Armando Martínez Cruz, José Tuxpan Vargas, José Alfredo Ramos Leal, Janete Morán Ramírez

**Affiliations:** ^1^Instituto Potosino de Investigación Científica y Tecnológica, A.C. División de Geociencias Aplicadas, San Luis Potosí, Mexico; ^2^CONAHCYT-Instituto Potosino de Investigación Científica y Tecnológica, A.C. División de Geociencias Aplicadas, San Luis Potosí, Mexico; ^3^CONAHCYT-Centro de Investigación en Materiales Avanzados, Durango, Mexico

**Keywords:** artificial intelligence, data science, Deep Learning, Machine Learning, Mexico, state-of-the-art

## Abstract

This systematic review provides a state-of-art of Artificial Intelligence (AI) models such as Machine Learning (ML) and Deep Learning (DL) development and its applications in Mexico in diverse fields. These models are recognized as powerful tools in many fields due to their capability to carry out several tasks such as forecasting, image classification, recognition, natural language processing, machine translation, etc. This review article aimed to provide comprehensive information on the Machine Learning and Deep Learning algorithms applied in Mexico. A total of 120 original research papers were included and details such as trends in publication, spatial location, institutions, publishing issues, subject areas, algorithms applied, and performance metrics were discussed. Furthermore, future directions and opportunities are presented. A total of 15 subject areas were identified, where Social Sciences and Medicine were the main application areas. It observed that Artificial Neural Networks (ANN) models were preferred, probably due to their capability to learn and model non-linear and complex relationships in addition to other popular models such as Random Forest (RF) and Support Vector Machines (SVM). It identified that the selection and application of the algorithms rely on the study objective and the data patterns. Regarding the performance metrics applied, accuracy and recall were the most employed. This paper could assist the readers in understanding the several Machine Learning and Deep Learning techniques used and their subject area of application in the Artificial Intelligence field in the country. Moreover, the study could provide significant knowledge in the development and implementation of a national AI strategy, according to country needs.

## 1 Introduction

Huge amounts of data are produced every day and extracting its information is essential to predict, interpret and create various smart applications in several fields, such as science, healthcare, education, financial modeling, policy, marketing, etc. (Sarker, 2021; Thrun et al., 2021; Zheng et al., 2024). Therefore, data management tools and techniques for advanced analysis that can extract insights and useful knowledge from vast data are needed.

In recent years, the acceleration of technological progress and the increase in computing capacity has increased, giving rise to the well-known fourth industrial revolution (Bughin et al., 2017; Velarde, 2019; Borges et al., 2021). In this sense, Artificial Intelligence (AI) development and its real-world applications have gained popularity due to the high results in terms of accuracy and efficiency, even surpassing humans’ performance (Angelov et al., 2021).

**Figure 1:**
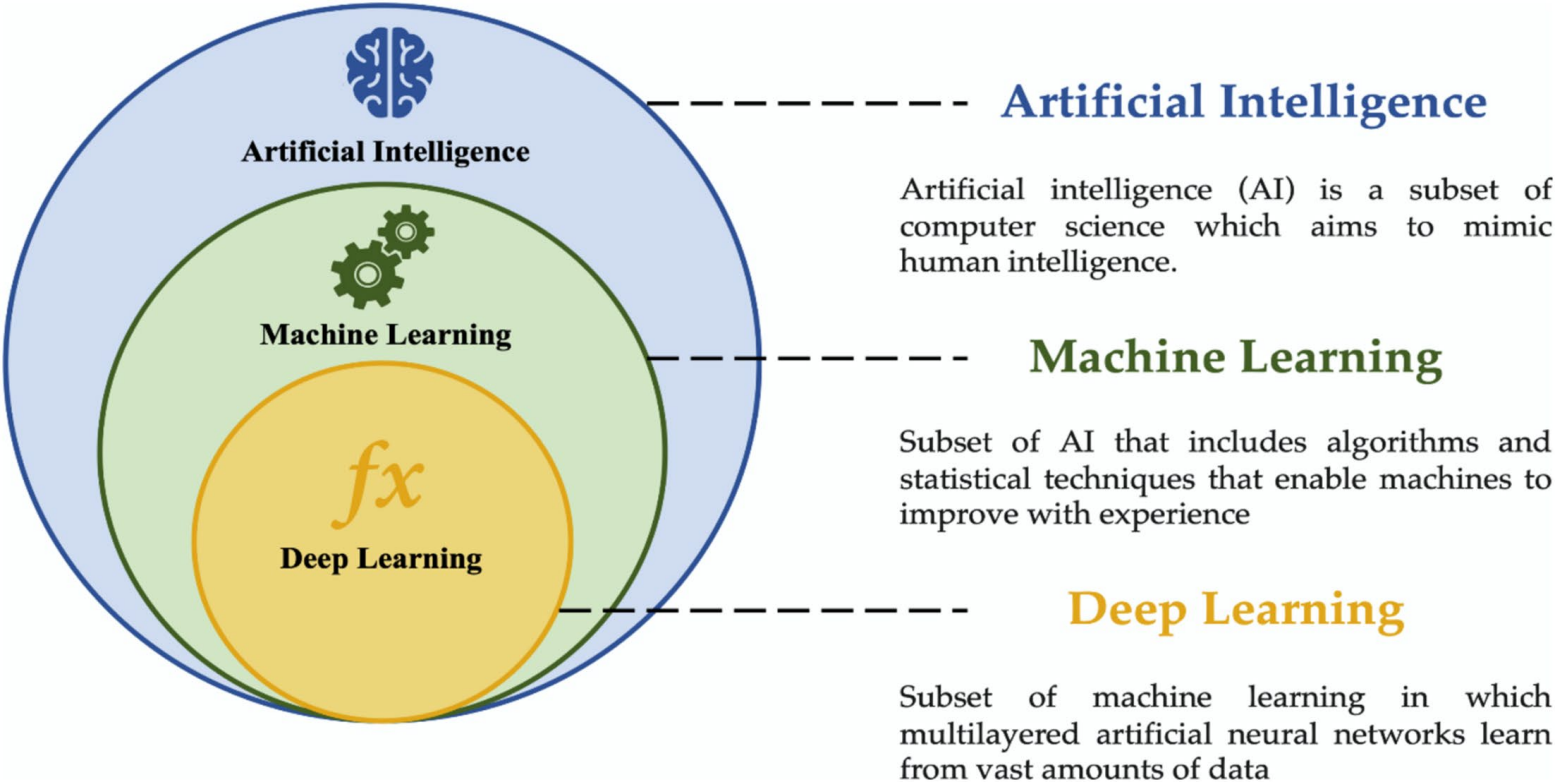
Brief definitions of artificial intelligence, machine learning and deep learning concepts.

Within that field, AI tools such as Machine Learning (ML) and Deep Learning (DL) algorithms have been capable of processing huge datasets efficiently, given that help to save time and maximize computing tools (Emmert-Streib, 2021; Xu et al., 2021). Sometimes the terms AI, ML, and DL are used as synonyms since they are closely related; however, it is important to distinguish the difference between them. Figure 1 shows the overview of these terms, further information can be consulted in specialized literature (Goodfellow et al., 2016; Mohammed et al., 2016; Chollet, 2021; Sarker, 2021).

**Figure 2:**
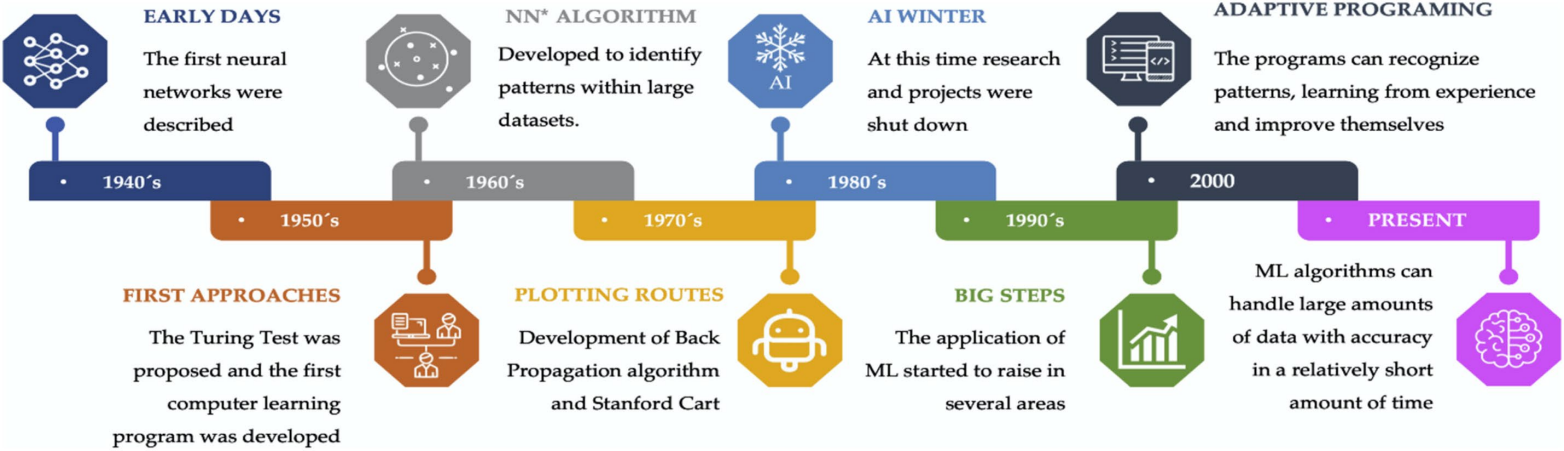
Timeline chart of some relevant events in ML and DL history.

Over time, there were key events that have led to the development of artificial intelligence, as shown in Figure 2. Several ML and DL algorithms were developed (e.g., Random Forest, Support Vector Machines, Neural Networks, KNN, etc.), and depending on the nature of the task, there are different approaches based on the type and volume of the data.

ML is a subfield of AI that allows computers to perform and improve tasks without explicit programming, with the development of algorithms and statistical tools (Jordan and Mitchell, 2015). In general, ML techniques can be classified into four groups (Mohammed et al., 2016; Hurwitz and Kirsch, 2018; Jhaveri et al., 2022):

- **Supervised learning:** usually starts with a pre-existing set of data and a pre-existing understanding of how that data is classified (labeled). This approach is intended to find patterns in data that could be applied in the analytical process. The algorithms are trained using preprocessed data, and the performance of the algorithms is evaluated with test data.
- **Unsupervised learning:** this approach is best suited when the task requires a massive amount of data that is unclassified (unlabeled), and the aim is to find a hidden structure in this data. The unsupervised algorithms segment data into clusters.
- **Semi-supervised learning:** in this type of learning, the data provided is a mixture of classified and unclassified data. This combination of labeled and unlabeled data is used to generate a suitable model for classifying the data.
- **Reinforcement learning:** this approach is a behavioral learning model in which the algorithm receives feedback from data analysis and guides the user to the best outcome. Because the system is not trained using the sample data set, reinforcement learning differs from other approaches.

Meanwhile, modern learning techniques have been coupled with Deep Learning architectures such as transfer learning and adversarial learning (Emmert-Streib, 2021). This since ML finds it difficult to the optimal combination of hyperparameters, extracted features, and pre-processing methods from a dataset. While DL approaches employ hierarchical layers to assemble levels of abstraction and model complex systems (Polson and Sokolov, 2020; Siddiqui et al., 2022).

DL is a branch of ML that applies Artificial Neural Networks (ANNs) that are characterized by numerous hidden layers. DL algorithms are commonly applied in pattern recognition systems, due to DL can be able to select optimal attributes for raw datasets (Alves de Oliveira and Bollen, 2023). DL has been applied and coped successfully with the high dimensional, noisy, and unstructured dataset (Janiesch et al., 2021). Moreover, DL has been widely applied in several areas of knowledge such as health issues (Pirovano et al., 2021), hydrological research (Chen et al., 2023), natural sciences (Garcke and Roscher, 2023), safety, road survey, and bridge inspection (Xu et al., 2021). In addition, outlier detection with Deep Learning methods such as reconstruction error, predictive error, and dissimilarity (Smejkalová et al., 2023).

In this current age of *big data*, ML and DL have become popular because of their learning capabilities from the past and their ability to make intelligent decisions (Sarker, 2021). Worldwide, exponential growth can be observed from several fields such as environmental science (Ardabili et al., 2020; Dokic et al., 2020; Saha et al., 2022; Ahmadi et al., 2022; Tao et al., 2022), medicine (Zhang, 2017; Valliani et al., 2019; Huang et al., 2020; Lui et al., 2020) or financial market (Warin and Stojkov, 2021; Ahmed et al., 2022). According to bibliometric studies, the United States of America (USA) and China are the leading countries in AI research, followed by Germany, the United Kingdom, India, Canada, and France (Rincon-Patino et al., 2018; Savage, 2020).

**Figure 3:**
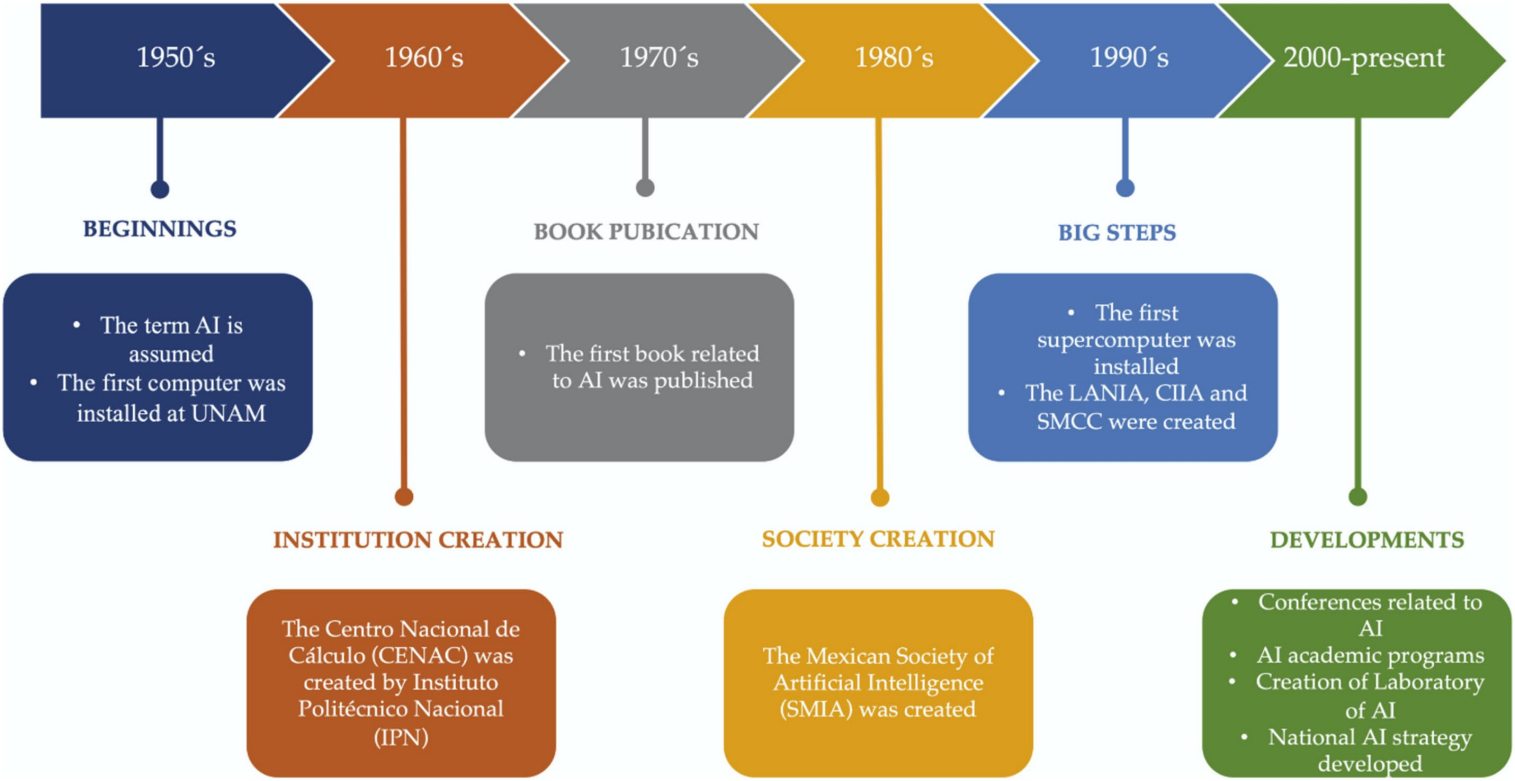
Timeline chart of some relevant events in ML and DL history.

Particularly, in Mexico AI has gained attention in the last two decades, Figure 3 shows the timeline chart of some main events regarding AI in Mexico, based on the report by Villegas-Vergara et al. (2021). Most of the relevant events were the creation of research centers and societies related to AI such as Centro Nacional de Cálculo (CENAC), Mexican Society of Artificial Intelligences (SMIA), Laboratorio Nacional de Informática Avanzada, A.C. (LANIA), Centro de Investigación en Inteligencia Artificial (CIIA) and the Sociedad Mexicana de Ciencia de la Computación (SMCC). From the year 2000, international conferences in AI were presented as well as the inclusion of academic programs related to this field.

Although some developments have been carried out, the application of AI in Mexico continues in its early stages. According to the World Government AI Readiness Index 2022, published by Rogerson et al. (2022) from Oxford Insights (OI), Mexico was placed 62nd in the rankings out of 161 countries. In this report three pillars named (1) Government, (2) Technology Sector and (3) Data and Infrastructure were evaluated. In academic research areas, between the years 2002–2017, Mexico’s National Council of Humanities, Science, and Technology (CONAHCYT) supported about 144 projects related to AI, including the computer sciences, data and information sciences, electronics, and telecommunications the relevant disciplines (Martinho-Trustwell et al., 2018).

However, nowadays there is uncertainty about the academic research areas, algorithms employed, trends in publication, location, etc. about AI applications. To our knowledge, there is no systematic review that summarizes the state-of-the-art research applications of ML and DL in the country. The aim of this work is to examine the variety of ML and DL algorithms employed in Mexico country, using the PRISMA method. Under this general objective, the answers are searched for the following research questions (RQ):

- **RQ1:** What are the publication trends using ML and DL approaches over the years?

To answer this question, a graph had to be constructed and analyzed. Additionally, characteristics such as publishing issues, institutions, and spatial distributions of research were explored.

- **RQ2:** Which are the research areas of application?

To answer this question, the results of the systematic review needed to be synthesized comprehensively, thus each paper was classified into a subject area based on its aim and scope.

- **RQ3:** Which ML and DL algorithms have been employed?

To search for answers to this question, the algorithms employed in each paper were identified. The results were included in a graph. Additionally, performance metrics issues were discussed.

The results obtained from this systematic review will allow us to identify gaps, challenges, and opportunities within this field. Furthermore, the information presented in this work could contribute to the planning, development, and improvement of the strategy in the national application of AI.

## 2 Methods

This systematic review provides a state-of-art of Machine Learning and Deep Learning development and its applications in Mexico in diverse fields. Through a rigorous and transparent method to minimize bias, researchers could identify gaps, trends, challenges, and opportunities, making evidence more accessible to decision-makers and guiding practice (Needleman, 2003; Paez, 2017). In this section, the detailed procedure employed in this systematic review is addressed.

### 2.1 PRISMA procedure

**Figure 4:**
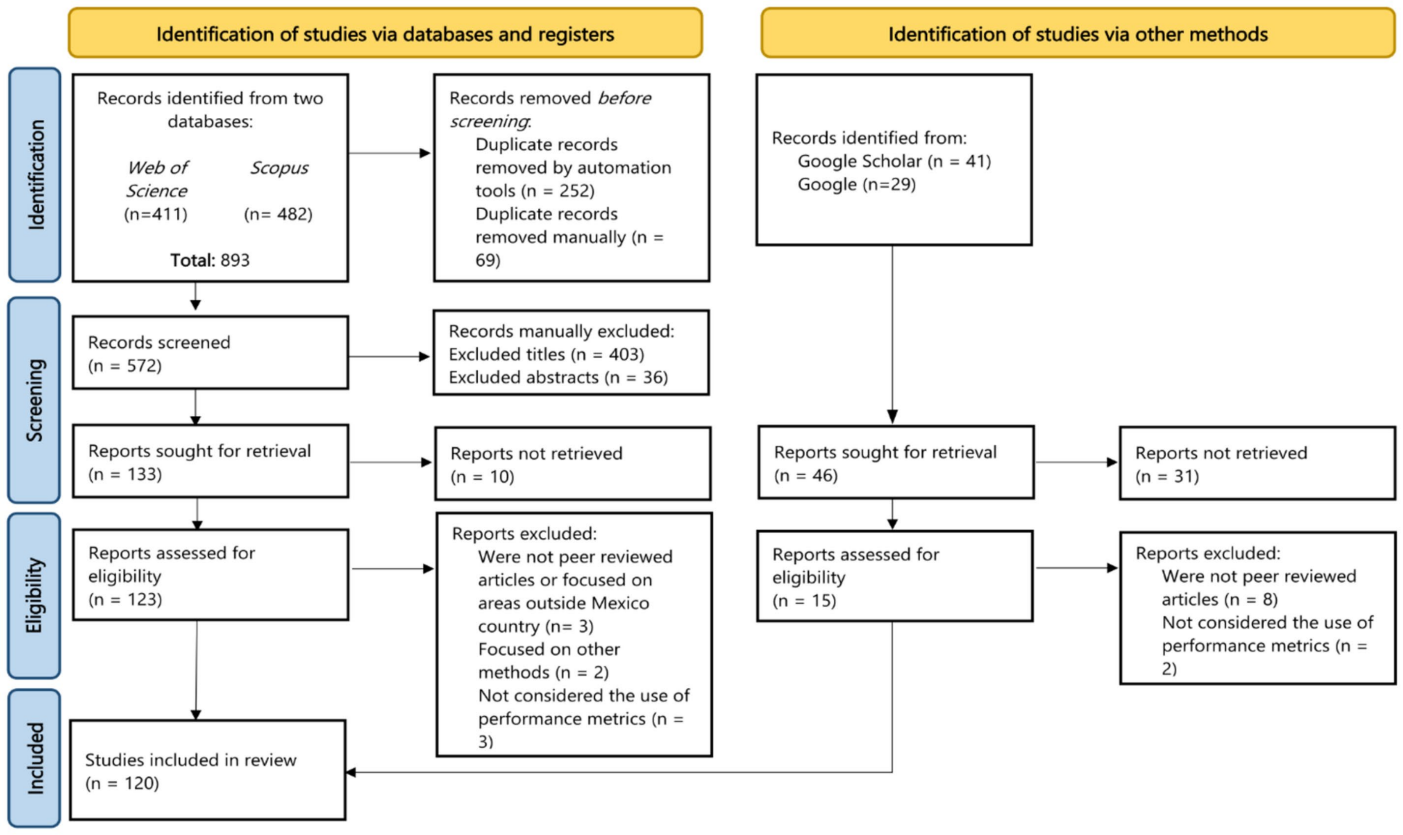
PRISMA flow diagram of this systematic review.

This systematic review was conducted using the PRISMA 2020 (Preferred Reporting Items for Systematic Reviews and Meta-Analyses) methodology, proposed by Page et al. (2021). This method consists of four steps namely: (1) identification, (2) screening, (3) eligibility, and (4) inclusion. Figure 4 shows the PRISMA flow diagram employed in this study.

#### 2.1.1 Identification

The present study utilized two of the most common databases, Web of Science and Scopus, accessed via the Instituto Potosino de Investigación Científica y Tecnológica A.C. Both databases are recognized as significant reliable sources of high-quality publications (Peixoto et al., 2021). The first stage of this search strategy consisted of data retrieval from databases by entering a search string, which included words such as “Artificial Intelligence,” “Machine Learning,” “Deep Learning” and “Mexico” (Table 1). The survey was conducted from the year 2000–2023, and the search was carried out on June 21st, 2023.

**Table 1:** Search string used in this systematic review.

Database	Search string
Scopus	TITLE-ABS-KEY((MACHINE LEARNING AND MEXICO) OR (DEEP LEARNING AND MEXICO) OR (ARTIFICIAL INTELLIGENCE AND MEXICO)) AND DOCTYPE(ar) AND SRCTYPE(j) AND (LIMIT-TO (PUBYEAR, 2023) OR LIMIT-TO (PUBYEAR, 2022) OR LIMIT-TO (PUBYEAR, 2021) OR LIMIT-TO (PUBYEAR, 2020) OR LIMIT-TO (PUBYEAR, 2019) OR LIMIT-TO (PUBYEAR, 2018) OR LIMIT-TO (PUBYEAR,2017) OR LIMIT-TO (PUBYEAR, 2016) OR LIMIT-TO (PUBYEAR, 2015) OR LIMIT-TO (PUBYEAR, 2014) OR LIMIT-TO (PUBYEAR, 2013) OR LIMIT-TO (PUBYEAR, 2012) OR LIMIT-TO (PUBYEAR, 2011) OR LIMIT-TO (PUBYEAR,2010) OR LIMIT-TO (PUBYEAR, 2009) OR LIMIT-TO (PUBYEAR, 2008) OR LIMIT-TO (PUBYEAR, 2007) OR LIMIT-TO (PUBYEAR, 2006) OR LIMIT-TO (PUBYEAR, 2005) OR LIMIT-TO (PUBYEAR, 2004) OR LIMIT-TO (PUBYEAR, 2003) OR LIMIT-TO (PUBYEAR, 2002) OR LIMIT-TO (PUBYEAR, 2001) OR LIMIT-TO (PUBYEAR, 2000))
Web of Science	((TS = ((“Machine Learning” AND “Mexico”) OR (“Deep Learning” AND “Mexico”) OR (“Artificial Intelligence” AND “Mexico”))) AND DT = (Article)) AND PY = (2000–2023)

Furthermore, other publications were found through a search on Google and Google Scholar, based on the study’s aims and scope. It is worthy to mention that both English and Spanish manuscripts were included.

A total of 893 articles from both Scopus and Web of Science databases met the string search criteria. On the other hand, 70 studies were identified from Google and Google Scholar sources. All documents were stored in the reference manager software Mendeley. Since multiple sources were used, and there were duplicate articles, the removal of duplicates was carried out by the automatic removal process by Mendeley and then verified manually. After duplicate removal, 572 articles from Scopus and Web of Science were retained for the next step.

#### 2.1.2 Screening

The second stage is known as the screening process, whereby articles are included or excluded based on the criteria decided by researchers. Remained articles were examined first by titles and then by abstracts. A total of 133 articles remained for retrieval from Scopus and Web of Science, where 10 articles were not retrieved in full-text. On the other hand, from Google and Google Scholar, 46 articles were sought for retrieval, whereas 31 papers were not retrieved.

#### 2.1.3 Eligibility

The third step of this procedure is eligibility, where full-text articles are assessed to include or exclude them based on the next inclusion criteria:

- The article should be an original research and present a study case in Mexico country. Technical reports, conference papers, and preprints will excluded.
- The article must train and employ a ML and/or DL algorithm for a specific purpose.
- The article should include quantitative performance metrics (if applicable) to report on its accuracy of prediction or significant differences of the models.

In this step, two reviewers accurately assessed the manuscript to determine which articles would be included. If both reviewers agreed to include a record, the agreement constituted the final decision. However, in cases of controversy, a third reviewer examined the manuscript and took the final decision. The detailed selection process based on inclusion criteria is presented in the Supplementary Excel data sheet (Supplementary Table S1).

#### 2.1.4 Inclusion

At this final stage, a total of 120 articles were included in this systematic review and investigated for meta-analysis using software such as MS Excel and QGIS v. 3.28.9. Details such as trends in publication, study location, research area, and algorithms employed, among others, were discussed.

## 3 Results and discussion

### 3.1 General overview

**Figure 5:**
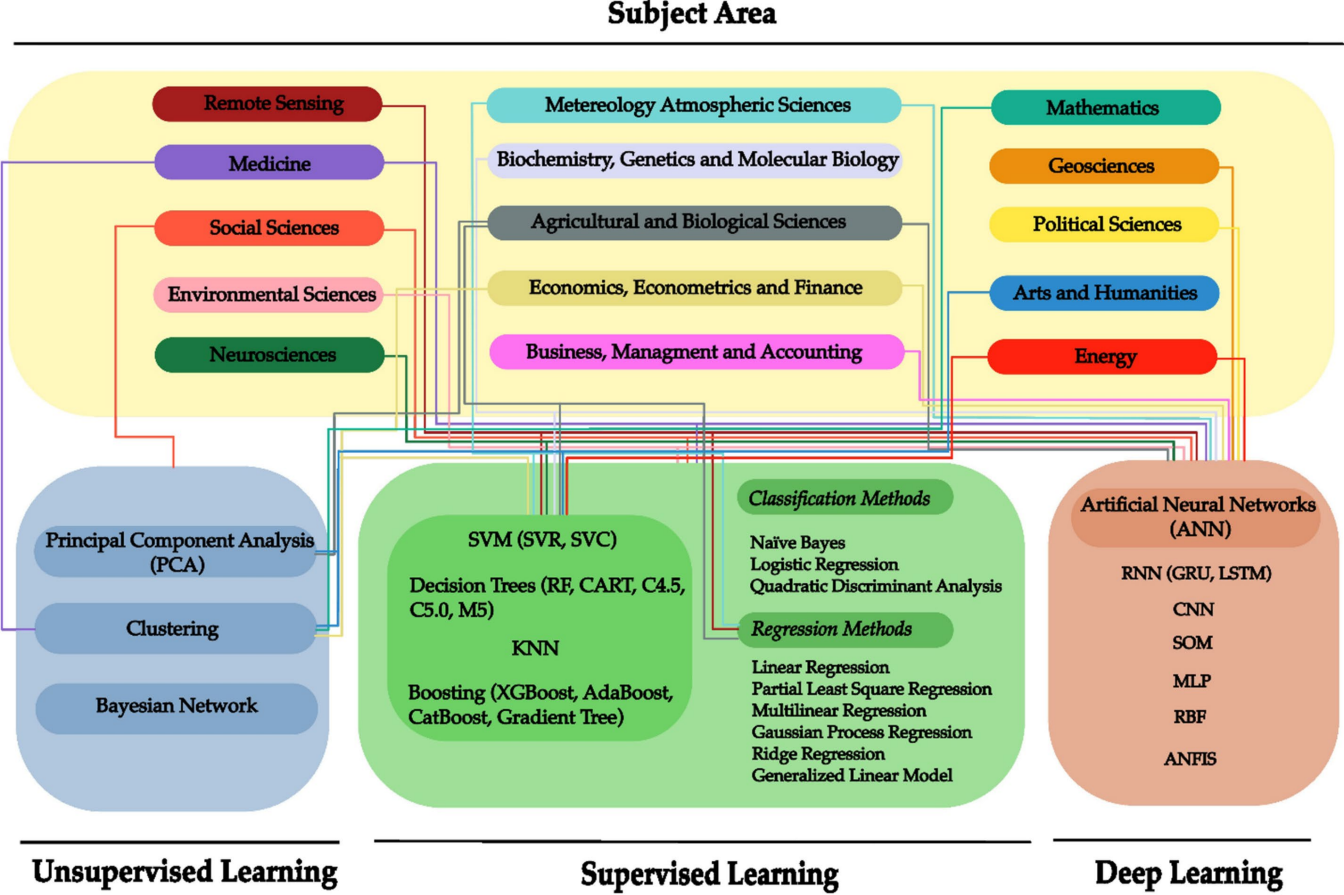
Reviewed algorithms and their subject area of application in Mexico.

A systematic review is critical for assessing and evaluating established literature and providing a comprehensive overview that might assist interested readers. Figure 5 outlines the reviewed ML and DL algorithms and their area of applicability. The extracted insights such as authors, location, algorithms, and performance metrics of the 120 research articles can be found in Supplementary Table S2.

### 3.2 Trends in publications

**Figure 6:**
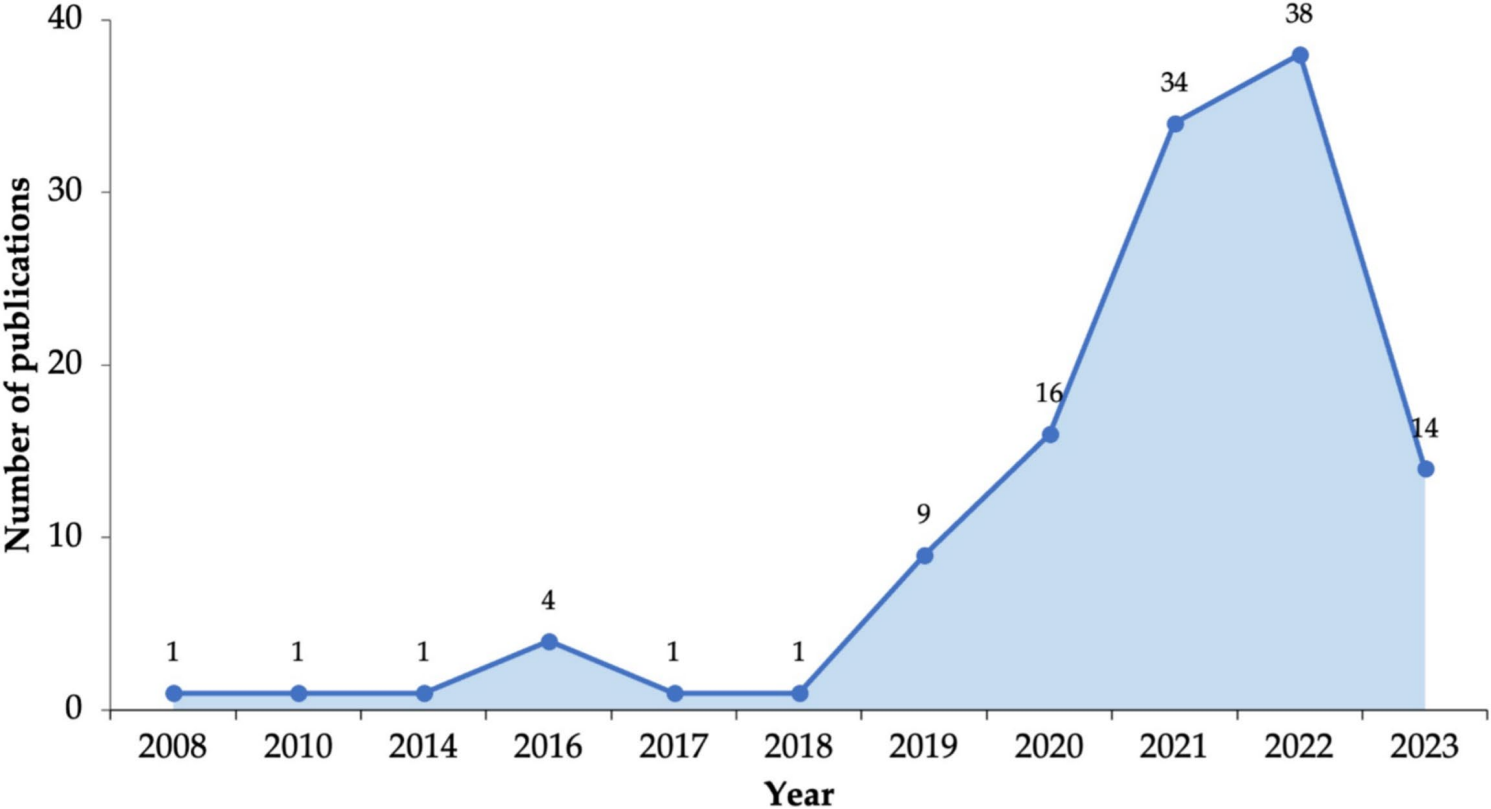
Publication trends on ML and DL in Mexico.

Regarding the RQ1, an analysis of Figure 6 was carried out. In the last 5 years, publications on ML and DL approaches in Mexico have grown up. The publication trends show that research dates back from 2008 and continued with few publications until 2018, with gaps in 2009, 2011, 2012, 2013, and 2015; thus, the publications were scarce over a decade. Research began to gain traction in 2019 (*n* = 9), showing an increase in 2020 (*n* = 16) and two peaks in 2021 (*n* = 34) and 2022 (*n* = 38). Until June 2023, 14 publications were recorded. Meanwhile, other countries around the world, such as China, the USA, the United Kingdom, and India have shown an exponential increase in the publication trends for artificial intelligence articles, during the period from 1991 to 2020 (Liu et al., 2021). For example, China has contributed by approximately 45% to the total number of articles published, while the USA and United Kingdom have maintained about 20 and 7% of worldwide article outputs, respectively. Other countries such as Canada, Germany, France, Italy, and Spain each now have contributed about 4% of the total global (Liu et al., 2021).

It is worth mentioning, that between 2002 and 2017, Mexico’s National Council of Humanities, Science and Technology (CONAHCYT) supported 144 projects related to AI (Martinho-Trustwell et al., 2018). However, in this review scarce research was found during that period, probably due to the application outside the research sector.

### 3.3 Publishing issues

**Figure 7:**
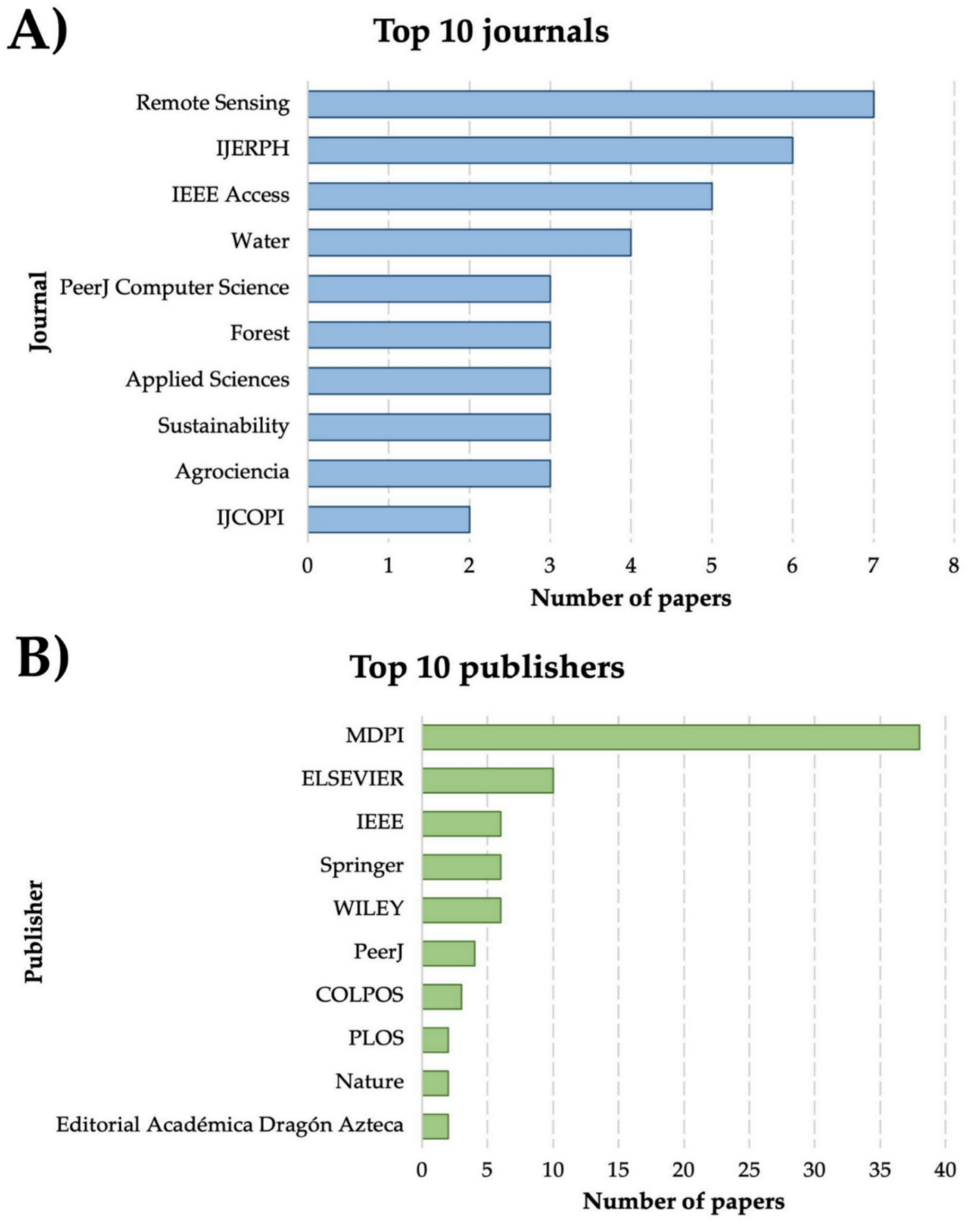
Publishing characteristics of the reviewed articles. **(A)** Journals. **(B)** Editorials.

Included records were published in a total of 84 different journals. Figure 7A shows the top ten journals, where Remote Sensing had the highest number of publications with seven records, followed by the International Journal of Environmental Research and Public Health (IJERPH) (*n* = 6), IEEE Access (*n* = 5) and Water (*n* = 4), remained journals have equal or less than 3 publications. In addition, based on our findings, a total of 41 different publishers were registered. Figure 7B shows the top 10 publishers, where the Multidisciplinary Digital Publishing Institute (MDPI) was the editor with the highest number of publications (*n* = 38), representing 33.63% of the total. Followed by ELSEVIER with 10 publications (8.85%) and WILEY, Springer, and IEEE with 6 (5.31%) each. The remaining editorials have equal or less than four publications.

### 3.4 Country and institutions

**Figure 8:**
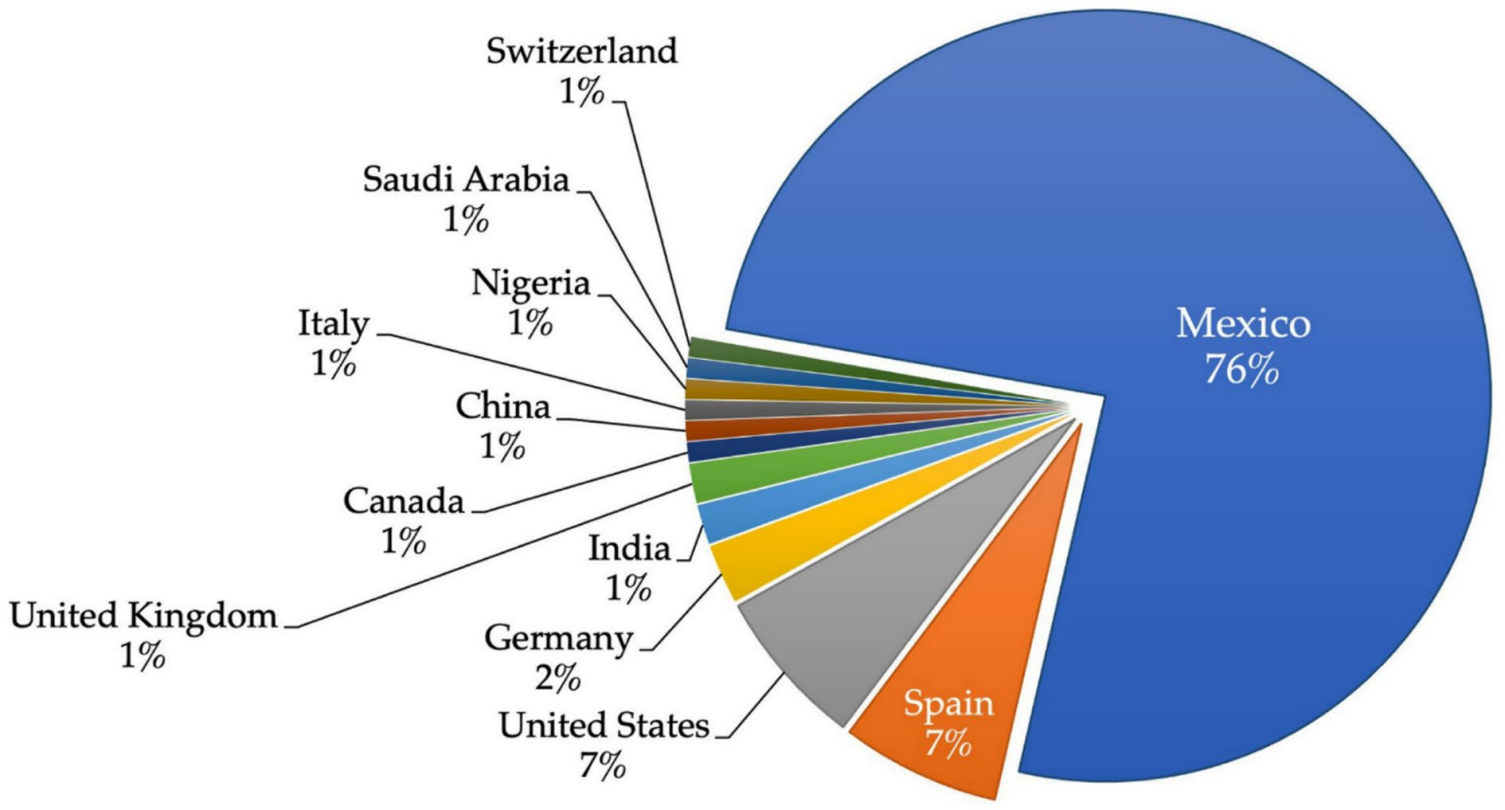
Spatial distribution by country, based on corresponding author affiliation.

For the country and institution representation, the data affiliation of the corresponding authors was extracted. A total of 12 countries have developed works related to ML and DL in Mexican territory (Figure 8). Mexico had the most corresponding authors with 76%, followed by Spain (7%), the United States (7%) and Germany (2%). The remaining countries have 1%.

**Figure 9:**
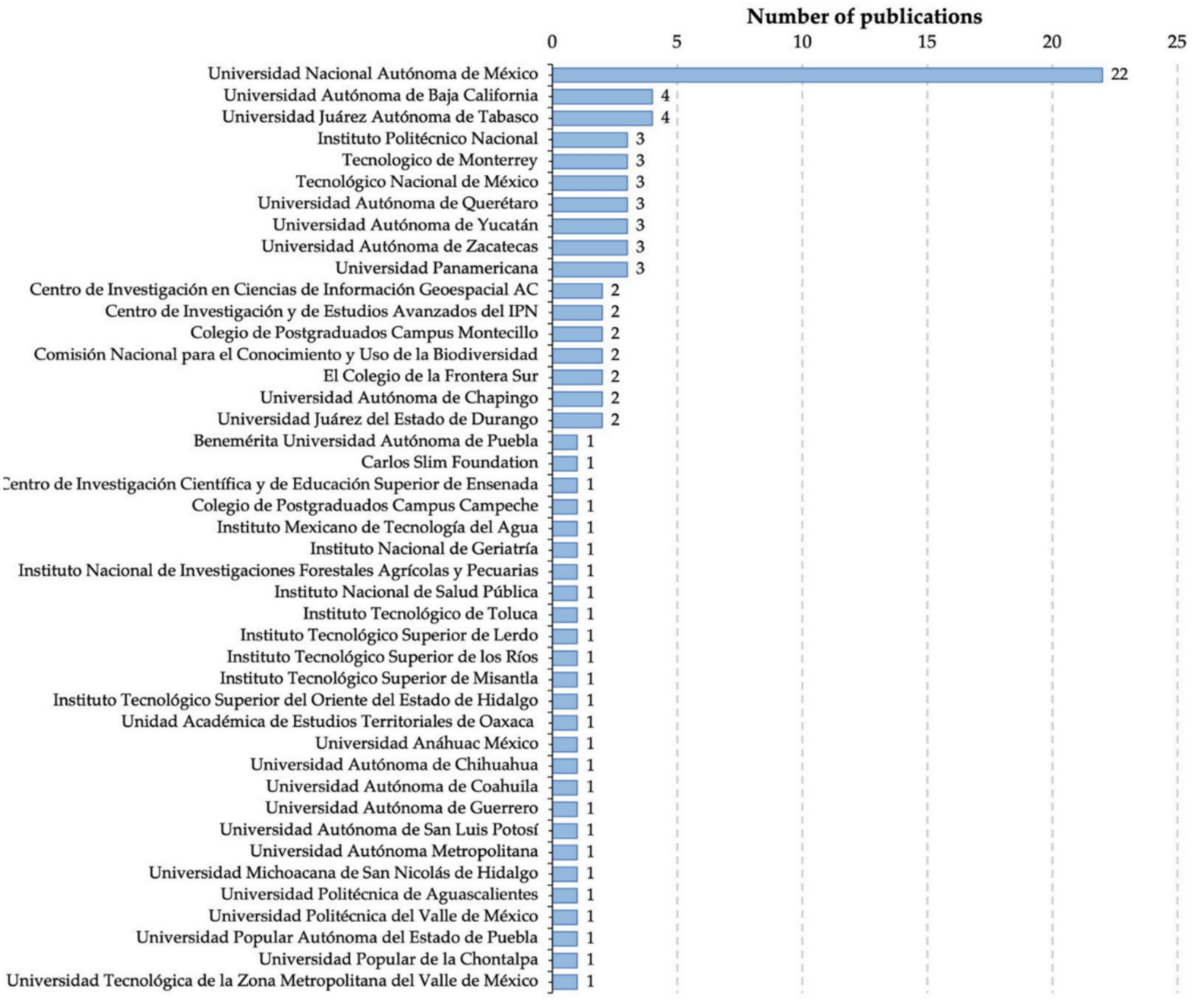
Mexican institutions that published articles related to topic.

Within Mexico country, a total of 43 institutions have published works on the field are shown in Figure 9. The Universidad Nacional Autónoma de México has most of the publications with 22. It is worthy mentioning that 14 of the publications in this institution belong to the author Salas-Rueda, who has been working on the application of Machine Learning techniques in the social field. The Universidad Autónoma de Baja California and the Universidad Juárez Autónoma de Tabasco have both four publications. Followed by the Instituto Politécnico Nacional, Tecnológico de Monterrey, Tecnológico Nacional de Mexico, Universidad Autónoma de Querétaro, Universidad Autónoma de Yucatán and Universidad Autónoma de Zacatecas with three publications all of them. The rest of the institutions have equal or less than two publications.

Meanwhile, other countries worldwide such as China, Singapore, the USA, the United Kingdom, and Iran have an important presence of universities and institutes that produced a large volume of artificial intelligence papers published between 1991 and 2020 (Liu et al., 2021).

### 3.5 Spatial distribution of the reviewed articles

**Figure 10:**
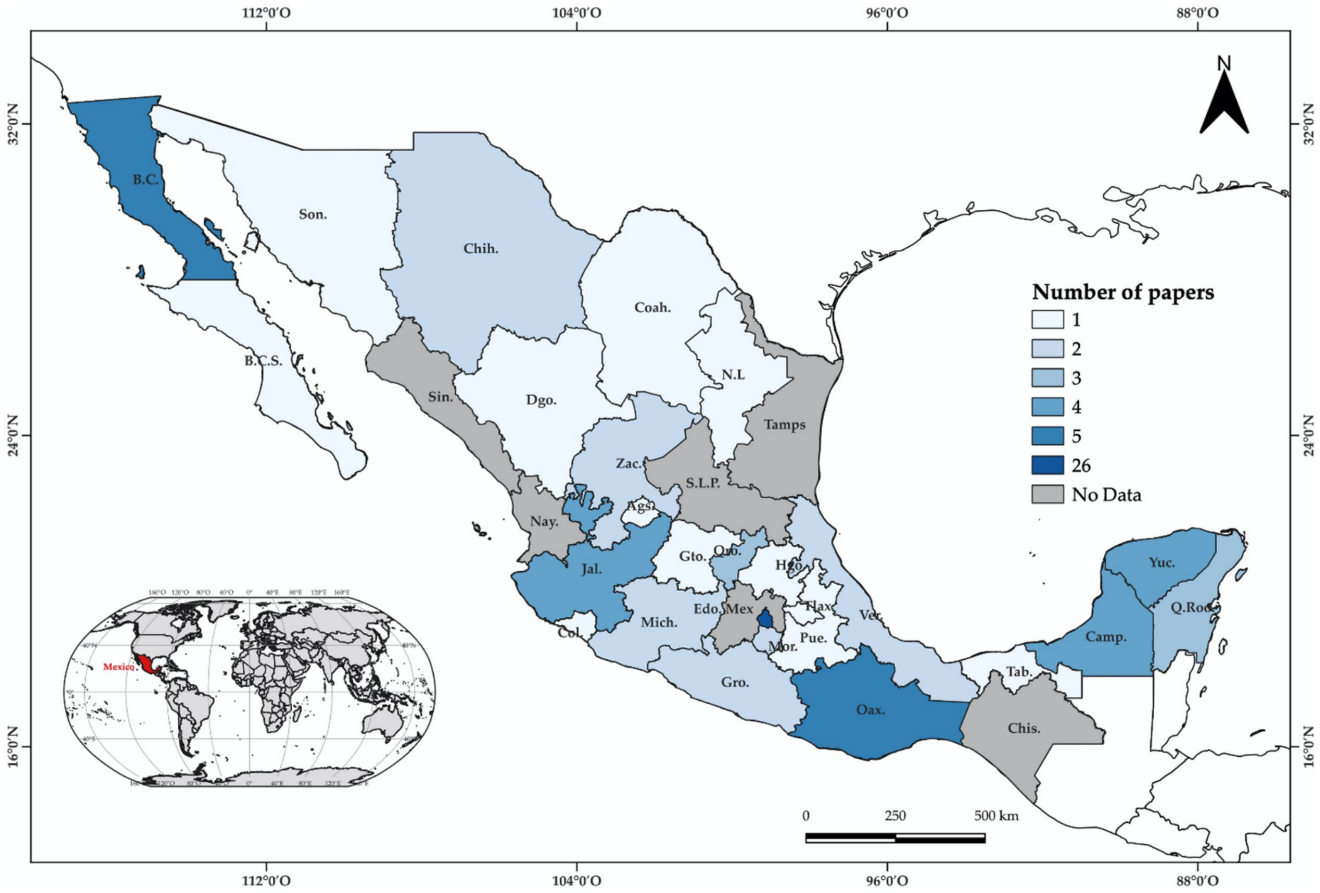
Spatial distribution by State of the reviewed articles (*n* = 78).

According to our findings, 49 articles (39%) were conducted at a national scale. These publications employed national databases mainly related to medical and social issues. On the other hand, the remaining research articles (*n* = 78) were conducted at a regional-local scale and their spatial distribution is shown in Figure 10. It was found that almost all the Mexican territory presents at least one publication; however, scientific production could be considered skewed. Most of the studies were concentrated in the center of the country at CDMX (*n* = 26). These findings agree with the report by Martinho-Trustwell et al. (2018), where the regional distribution of related research is biased, with the great majority of academic production occurring in CDMX.

Meanwhile, the southeast region was represented by Quintana Roo (Q.Roo) (*n* = 3), Yucatán (Yuc.) (*n* = 4), and Campeche (Camp.) (*n* = 4) whereas the southwest region by Oaxaca with five publications. The northwest region was represented by Baja California (B.C.) with also five publications. The states of Chiapas (Chis.), Estado the México (Edo.Mex.), Nayarit (Nay.), San Luis Potosí (S.L.P.), Sinaloa (Sin.), and Tamaulipas (Tamps.) did not present research related to the topic.

In a previous study for South American countries, the total number of artificial intelligence papers published was appraised, using the Index Latin Artificial Intelligence (ILAI) where Mexico represented fourth place, below countries like Brazil, Chile, Ecuador, and Uruguay (UNESCO, 2024). This could be linked mainly to the backlog of their economy and artificial intelligence readiness (Rogerson et al., 2022).

### 3.6 Subject areas

**Figure 11:**
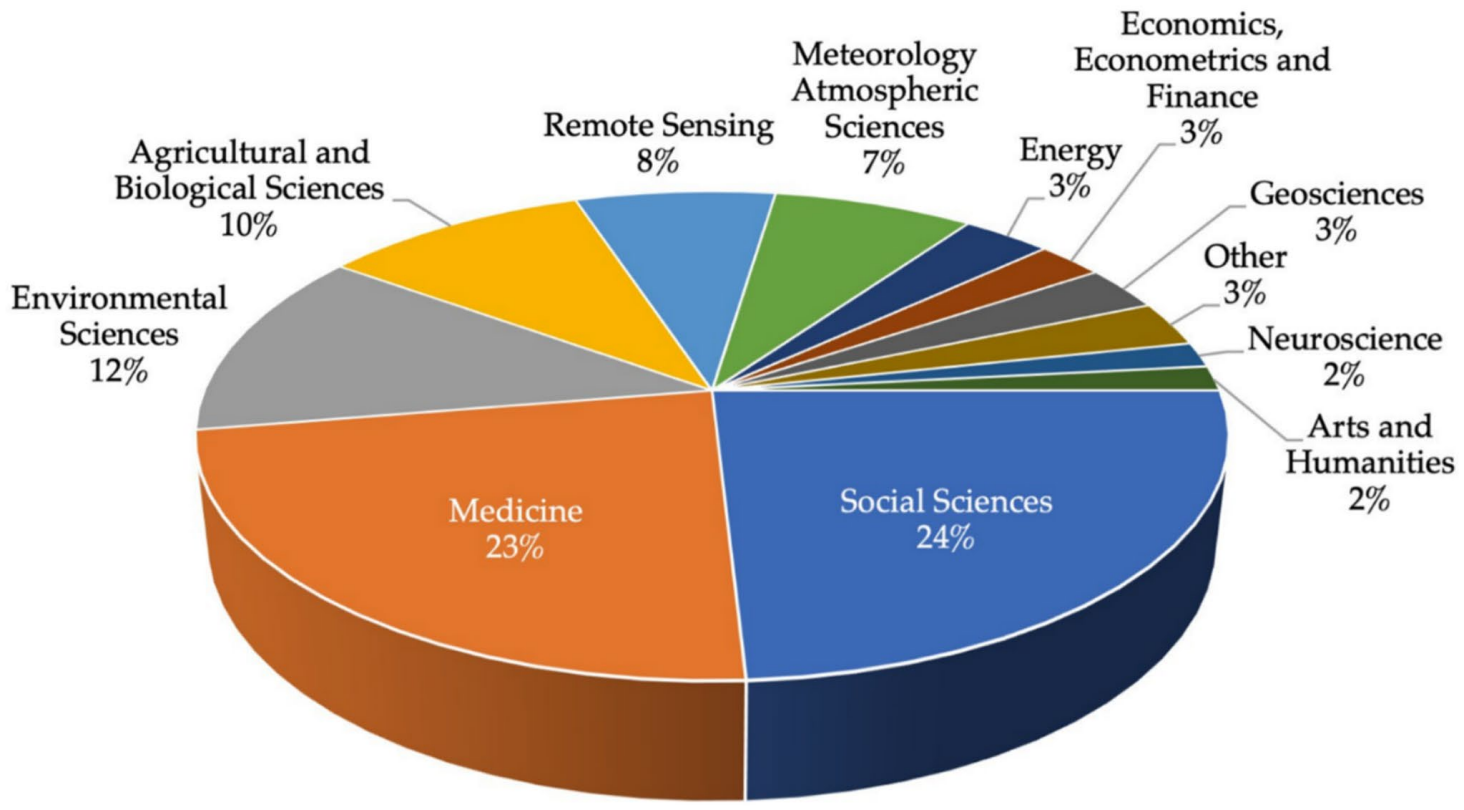
Percentage of subject areas in the reviewed research papers.

Over time, the literature has shown several application fields with Machine Learning and Deep Learning, which includes computer vision, healthcare, semantic analysis, social issues, and financial services, among others (Angra and Ahuja, 2017; Shinde and Shah, 2018; Ghahremani-Nahr et al., 2021; Sharma et al., 2021). The information obtained in this subsection allowed us to answer the RQ2. The surveyed articles were distributed in a total of 15 general subject areas, as shown in Figure 11. Most of the articles fall within the fields of social sciences and medicine, with 24 and 23%, respectively. Followed by Environmental Sciences (12%) and Agricultural and Biological Sciences (10%). In a minor percentage, there are the fields of neuroscience and arts and humanities with both 2%.

Nowadays, social scientist lives in an era of big data where information is being produced from several sources (e.g., social media, websites, etc.). Thus, ML and DL tools are increasingly being utilized to extract meaningful information from these datasets (Grimmer, 2015; Grimmer et al., 2021). In the Social Sciences area, some research has been focused on social networks to identify gender-based violence (Gutiérrez-Esparza et al., 2019; Castorena et al., 2021), sentiments during COVID-19 (Corona, 2022; Contreras-Hernández et al., 2023), among others. Meanwhile, other studies were focused on teachers’ and students’ perception about the use of educational web applications and Information and Communications Technology (ICT) (Salas-Rueda, 2020; Salas-Rueda et al., 2020b, 2020a, 2021d, 2021b, 2021e, 2021a, 2021c, 2022a, 2022c, 2022b, 2022d; Salas-Rueda and Castañeda-Martínez, 2021; Salas-Rueda and Ramírez-Ortega, 2021).

Regarding Medicine area, both AI approaches have been widely applied in the medical field (medical imaging, brain issues, cancer diagnosis, etc.), showing an enhancement of performance and reliability in comparison with traditional methods (Bakator and Radosav, 2018; Miotto et al., 2018; Shehab et al., 2022b). During the last two years, in Mexico various ML and DL approaches have been applied to address the problems that have arisen due to the COVID-19 pandemic (Almustafa, 2021; Castillo-Olea et al., 2021; Chadaga et al., 2021; Guzmán-Torres et al., 2021; Muhammad et al., 2021; Quiroz-Juárez et al., 2021; Becerra-Sánchez et al., 2022; Pradhan et al., 2022; Prieto, 2022; Rojas-García et al., 2023). On the other hand, studies have been focused on the study sarcopenia process (Castillo-Olea et al., 2019, 2020; Carrillo-Vega et al., 2022) as well as metabolic syndrome (Gutiérrez-Esparza et al., 2020, 2021). Detailed information regarding the reviewed articles within their corresponding subject areas is shown in Supplementary Table S1.

According to Maslej et al. (2024) the total number of artificial intelligence publications worldwide, by field study in the period from 2010 to 2022 is ML which has increased sevenfold since 2015 with 72,230 AI publications, computer vision (21,309 papers), pattern recognition (19,841papers), and process management (12,052 papers).

### 3.7 Algorithms employed

**Figure 12:**
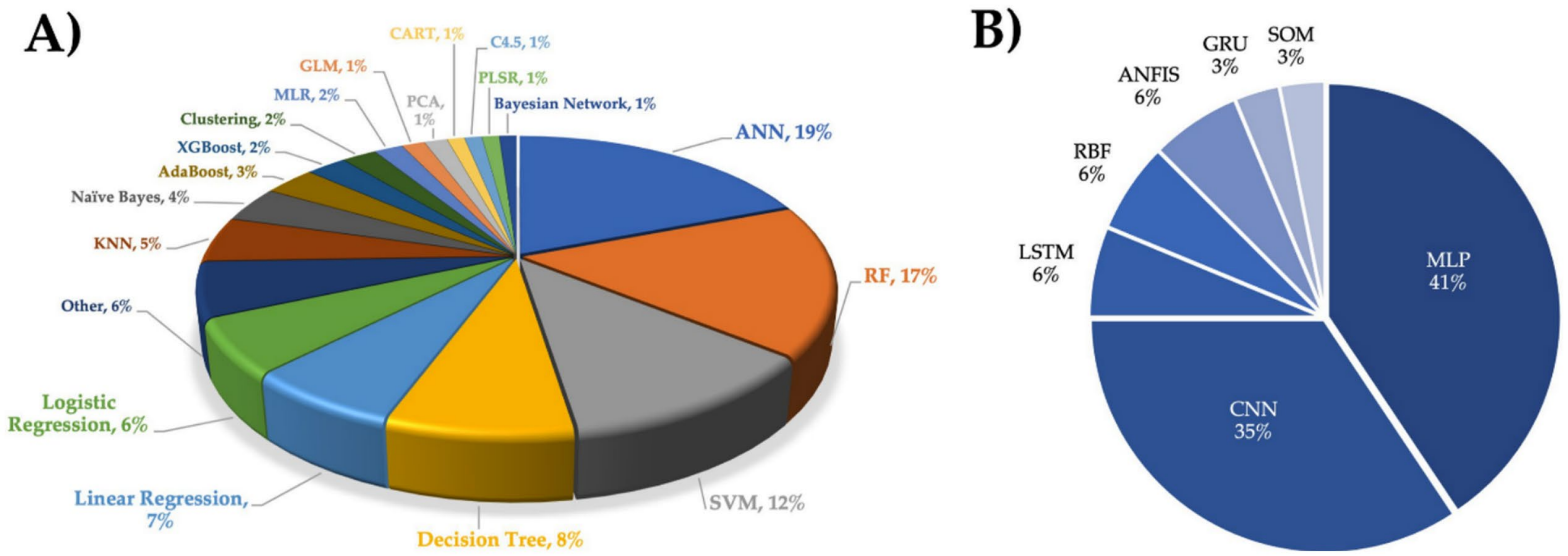
Applied algorithms in the reviewed articles. **(A)** Percentage of use. **(B)** Artificial Neural Network types.

Several ML and DL algorithms have been developed (Figure 12), and their efficacy is highly dependent on the integrity and quality of the input data (Alzubaidi et al., 2021).

Figure 12A presents the algorithm types that have been applied in Mexico country and allowed to respond to the RQ3. A total of 30 different algorithms were reported, where Artificial Neural Networks (ANN) were the most employed at 20%, followed by Random Forest (RF) at 17% and Support Vector Machines (SVM) at 12%. Other algorithms (6%) included Gradient Tree Boosting, CatBoost, M5 algorithm, and ridge regression, among others. ANN is a type of artificial intelligence that is inspired by a biological nervous system (Malekian and Chitsaz, 2021). The simplest ANN comprises a three-layer structure: input, hidden, and output layer, with connected neurons (nodes) to simulate the human brain. The existing nodes process and transmit input signals to the subsequent nodes, simulating the synapsis connections of the brain (Cui et al., 2020). This tool has become popular and powerful for classification, clustering, prediction, and pattern recognition due to its facility to model non-linear and complex or multi-complex tasks (Abiodun et al., 2018, 2019; Shehab et al., 2022a).

The ANN types employed in the reviewed articles are shown in Figure 12B. Multilayer Perceptron (MLP) was the most employed ANN with 41%, followed by Convolutional Neural Networks (CNN) with 35%. MLP is considered the preferred ANN due to its capacity to differentiate nonlinearly separable data and is trained using the backpropagation (BP) learning algorithm (Ramchoun et al., 2016; Mohseni-Dargah et al., 2022). The performance of MLP is determined not only by the input variables, number of hidden layers, nodes, and training data, but also by other parameters such as learning rate, momentum, and number of iterations (Taud and Mas, 2017). Meanwhile, CNN is a Deep Learning model that is inspired by the arrangement of the animal visual cortex and is used to analyze data with a grid pattern, such as images, being relevant to computer vision tasks (Yamashita et al., 2018; Li et al., 2022). This model is composed of convolution layers, pooling layers, and fully connected layers (Indolia et al., 2018; Yamashita et al., 2018).

In addition to traditional ML and DL models, Transformers have become popular and are used in various disciplines, including natural language processing, computer vision, and speech processing, due to their capacity to capture contextual relationships within sequential data (Vaswani et al., 2017; Zhang et al., 2023). Moreover, those models have also been adopted in other research areas, such as chemistry, life sciences, sentiment analysis, and health sciences (Lin et al., 2022; Thoyyibah et al., 2023). The results of this review showed that there have been no applications of Transformers in any reviewed work.

### 3.8 Performance metrics (PM’s)

**Figure 13:**
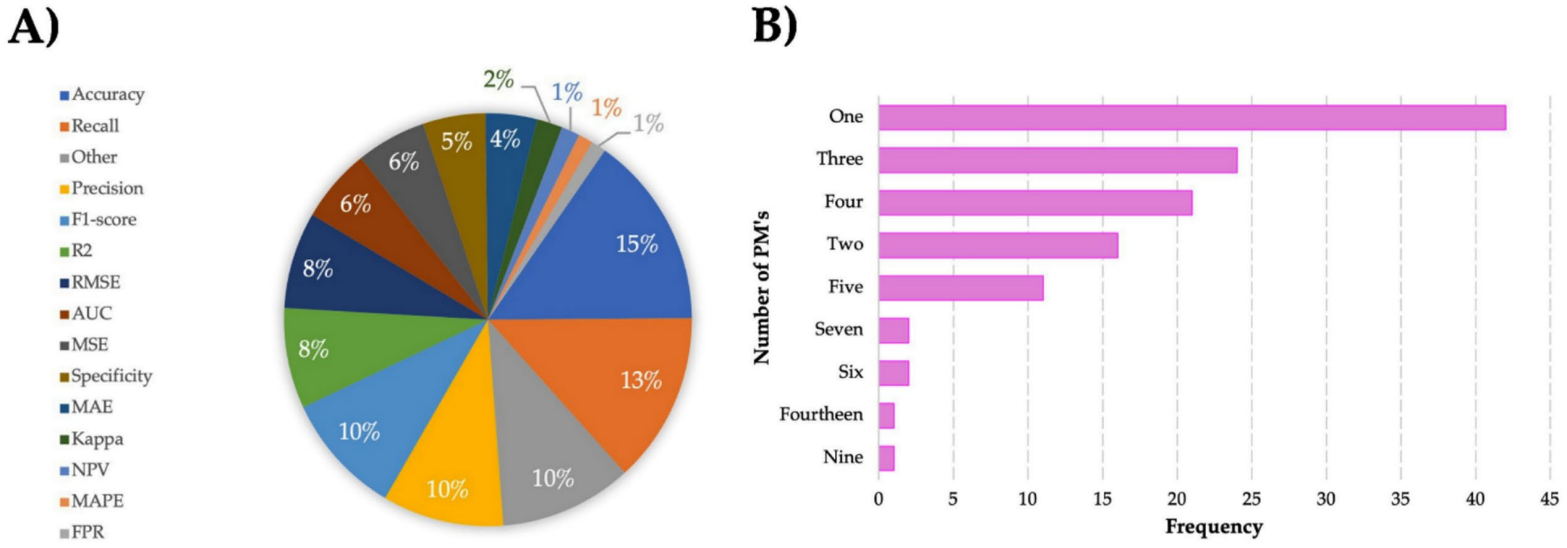
Analysis of applied performance metrics (PM’s). **(A)** Percentage distribution of individual PM’s. **(B)** Combination frequency of PM’s.

A performance metric can be defined as a logical and mathematical construct that describes and measures how close the actual results from what has been expected or predicted (Botchkarev, 2019; Karthik et al., 2023). A wide variety of PM’s has been proposed and used to evaluate the performance of AI models. For regression methods, the main metrics include R^2^, RMSE, and MSE. Meanwhile, for classification methods, accuracy, precision, and recall are some of the common metrics applied (Botchkarev, 2019; Erickson and Kitamura, 2021; Huang et al., 2021). Figure 13A presents the percentages of the used PM’s in the reviewed articles. Accuracy (15%) and Recall or Sensitivity (13%) were the most PM’s employed, followed by Precision and F1-score, both with 10%. Other PM’s (10%) encompass Nash-Sutcliffe Efficiency (NSE), Precision-Recall-Area, or correlation coefficient (*r*). Metrics such as the Kappa Index (2%) or the Mean Absolute Percentage Error (MAPE) (1%) were less frequent.

On the other hand, Figure 13B shows the combined frequency of PM’s. Among all studies, the use of one performance metric was preferred (*n* = 42), followed by three (*n* = 24) and four (*n* = 21). Even when there is no rule of how many PM’s to use, researchers are encouraged to employ various PM’s to make the performance evaluation more strong.

## 4 Limitations

Although this study was carried out by a systematic methodology, some limitations must be considered. The articles included in this review were retrieved from Web of Science and Scopus databases, through the electronic resources of the Instituto Potosino de Investigación Científica y Tecnológica A.C. Since we considered only two databases, there is a possible paper omission since other databases exist in both Spanish and English language, such as PubMed, IEEE, JSTOR, Redalyc, Scielo, among others. This study considered only journal articles to ensure that the included articles were of high quality and had undergone through a peer-review process. Technical reports, conference papers, and preprints were not included. Moreover, some manuscripts that meet the inclusion criteria were not retrieved in full text because the institution does not have full access to the content. Further reviews could also make modifications in the search string (e.g., adding the algorithm terms) to obtain more accurate results. The findings of this study provide essential information about the research panorama of AI in Mexico and its area applications, this type of work should continue to discover more gaps, challenges, and opportunities.

## 5 Future directions and opportunities

AI has developed sufficiently as a scientific discipline and technology, having extended from laboratories to the entire community. Nowadays, industry and government administrations are rapidly using intelligent and digital technologies in their daily tasks and undertakings, bearing in mind the Sustainable Development Goals (Vinuesa et al., 2020; Palomares et al., 2021). Research publications employing ML and DL approaches in Mexico have increased considerably in the last 5 years and are expected to continue rising, this could be an inflection point since some opportunities for development are present.

Based on the findings of this study, there is a potential to make a big scientific contribution by studying water resources using AI techniques. Worldwide, both ML and DL approaches have been applied to solve complex water-related problems, including real-time monitoring, forecasting, water resources allocation, water systems technology optimization, pollutant source identification, and pollutant concentration estimation (Huang et al., 2021; Zhu et al., 2022). Some applications in surface water included the development of water quality prediction and analysis (Wai et al., 2022; Zhu et al., 2022; Irwan et al., 2023), whereas in groundwater resources, predicted characteristics such as discharge, groundwater recharge, groundwater level fluctuation, aquifer loss coefficient, among others, has been studied by researches (Ahmadi et al., 2022). Mexico is confronted by several water difficulties, including water scarcity, pollution, and ineffective water administration. The modeling efforts mostly focused on general processes such as conflict resolution, water resources planning, water availability, and demand diagnosis, with the application of traditional software (e.g., Stella, UVQ, SWAT, MODFLOW, etc.) (Hernández-Cruz et al., 2022). In this research, a few published works related to the study of water resources were found, thus we encourage related researchers to focus on this area.

In some cases, the main limitations are the data acquisition and availability, which is a fundamental resource for AI models. Often data is inadequate and incomplete, or difficult to obtain through traditional *in-situ* methods. In this sense, remote sensing could provide essential information for data extraction, image classification, change detection, or accuracy assessment (Maxwell et al., 2018; Ma et al., 2019). Our research has demonstrated that published work related to remote sensing is scarce, with only 8% of the total reviewed papers; thus, there is an opportunity for development in this area.

Furthermore, where it is possible to collect high-quality data, advanced techniques such as Transformers can open the door to capturing temporal relationships in history for prediction or classification tasks, where they have been successfully applied in related works, even (Méndez et al., 2022; Maurício et al., 2023; Islam et al., 2024). Thus, we encourage you to explore this area.

Additionally, if there are no conflicts of interest or legal issues, Open-source AI models and data sharing are suggested as ways to enable rapid development and creation of new models. In Mexico, the National Digital Strategy encourages open data sharing through the https://datos.gob.mx/ platform.

Our findings in this study should not be generalized, since only provide academic scientific production. The application and use of AI tools in the country have taken place in big companies (e.g., industry, computer science, business, telecommunications) as well as in education at all levels with the implementation of new technologies (Martinho-Trustwell et al., 2018). However, toward a robust national AI strategy, transdisciplinary collaboration between academia, industry, and civil society is recommended. The results of this study could provide essential bases to continue the scientific production in the country, toward the development of guidelines for an AI strategy.

## 6 Conclusion

This work is the first approach to summarize the current state-of-the-art in the research panorama of ML and DL models within a developing country such as Mexico. A systematic methodology was conducted via the PRISMA 2020 statement. Summarizing the trends in publications provided the answer to RQ1. The publications in the country were scarce over a decade, having a significant increase in the last 5 years, with two peaks in 2021 and 2022. Furthermore, most of these studies have been carried out in the central zone of the country, showing a location bias. Forty-three institutions were identified with research publications, where the Universidad Autónoma Nacional de México presented more in comparison with the rest. The aim and scope of each paper allowed us to answer RQ2. A total of 15 subject areas were identified, where Social Sciences and Medicine were the main areas of applications, whereas areas such as Geosciences were less explored. Exploring the answer to RQ3 led to the identification that ANN models were preferred, probably due to their capability to learn and model non-linear and complex relationships. Other popular models included RF and SVM. In general terms, the selection and application of the algorithms rely on the study objective and the data patterns. Regarding the performance metrics applied, accuracy and recall were the most employed. This review presented a general panorama of the research publications in the AI field, this work will help readers to understand the several ML and DL techniques used and their subject area of application. Moreover, the study could provide significant knowledge in the development and implementation of a national AI strategy, according to country needs as well as encourage multidisciplinary and collaboration opportunities.
