# Supplementary material for: A systematic review of Machine Learning and Deep Learning approaches in Mexico: challenges and opportunities
Source: Front Artif Intell. 2025 Jan 7;7:1479855. doi: 10.3389/frai.2024.1479855 (PMC11753225; doi:10.3389/frai.2024.1479855)
Supplement: Supplementary file 2 [file Table_2.DOCX]

Supplementary Material

A systematic review of Machine Learning and Deep Learning approaches in Mexico: challenges and opportunities

José Luis Uc Castillo, Ana Elizabeth Marín Celestino*, Diego Armando Martínez Cruz, José Tuxpan Vargas, José Alfredo Ramos Leal and Janete Morán Ramírez

*** Correspondence:** Ana Elizabeth Marín Celestino, ana.marin@ipicyt.edu.mx

# Supplementary Tables

| **Research** | **Location** | **Applied models** | **Performance metrics** |
| --- | --- | --- | --- |
| Remote Sensing | | | |
| Arellano-Verdejo and Lazcano-Hernández (2021) | Campeche, Yucatán, Quintana Roo | CNN | Accuracy, Recall, Precision, F1-score |
| Arellano-Verdejo et al. (2019) | Quintana Roo | CNN, MLP | Accuracy |
| Arias-Rodriguez et al. (2020) | CDMX | Linear Regression, RF, SVR, GPR | RMSE, R^2^ |
| Carmona et al. (2021) | Nuevo León | MLR, ANN | *r*, RMSE, MAE |
| López-Serrano et al. (2020) | National | RF, SVR | RMSE, R^2^ |
| Mejia-Zuluaga et al. (2022) | CDMX | SVC | Accuracy, Recall, Precision, Kappa |
| Pech-May et al. (2022) | Tabasco | SVM, RF, CART | Accuracy, Kappa |
| Salvador et al. (2020) | National | SVM, RF, GLM | %RMSE, R^2^ |
| Schulthess et al. (2023) | Sonora | RF | Accuracy, Recall, Precision, F1-score |
| Meteorology Atmospherics Sciences | | | |
| Ahmad et al. (2022) | CDMX | RF, GTB, ANN, MLR | R^2^, Index of Agreement (IOA) |
| Becerra-Rico et al. (2020) | CDMX | GRU, LSTM | RMSE |
| Contreras-Navarro et al. (2016) | Baja California | ANN, PLSR, GLR, Ridge Regression, CART | nRMSE, R^2^, VAR |
| Coria et al. (2016) | National | C4.5 | Accuracy, F1-score, Kappa |
| Espinosa-Guzmán et al. (2017) | Campeche | ANN | R^2^, MSE, MAE |
| Gutiérrez-Avila et al. (2022) | CDMX | XGBoost | RMSE, MAE, MAD, R^2^, SD |
| Quej et al. (2022) | Campeche, Yucatán, Quintana Roo | SVM, ANFIS, CatBoost | R^2^, NSE, RMSE, MAE, MBE |
| Magallanes-Quintanar et al. (2023) | Zacatecas | ANN | R^2^, MSE, MAE |
| Ramos-Cirilo et al. (2021) | Campeche | SVM, XGBoost, GEP | R^2^, MAE, RMSE, MBE |
| Zhu and Aguilera (2021) | National | RF | RMSE, MAE, R^2^ |
| Medicine | | | |
| Almustafa (2021) | National | Naive Bayes, RF, KNN, SGD, Decision Tree | RMSE, MAE, Accuracy, AUC |
| Appice et al. (2020) | National | M5, SVR, KNN | RMSE |
| Argueta-Santillan et al. (2021) | National | CNN, SVM, RF, Logistic Regression, Naïve Bayes | Accuracy, Recall, Precision, F1-score |
| Baak-Baak et al. (2022) | National | Clustering | Kruskal-Wallis |
| Becerra-Sánchez et al. (2022) | National | KNN, Logistic Regression, RF, ANN | Accuracy, Recall, Precision, F1-score |
| Carrillo-Vega et al. (2022) | National | Self-organizing map (SOM) | Kruskal Wallis, ANOVA |
| Castillo-Olea et al. (2019) | Baja California | KNN, SVM, RBF, Gaussian Process, RF, MLP, AdaBoost, Naïve Bayes, QDA | Accuracy, Precision, F1-score |
| Castillo-Olea et al. (2020) | Baja California | SVM, Decision Tree, RBF, Gaussian Process, RF, MLP, AdaBoost, Naïve Bayes, QDA | Accuracy, Precision, F1-score |
| Castillo-Olea et al. (2021) | Baja California | Decision Tree, SVM, RF, Logistic Regression, MLP, Naïve Bayes, AdaBoost | AUC, Accuracy, F1-score, Precision, Recall |
| Chadaga et al. (2021) | National | RF, AdaBoost, XGBoost, Catboost | Accuracy, Precision, Recall, F1-score, AUC |
| Gallardo-Rincón et al. (2023) | National | ANN | Recall, Specificity, precision, AUC |
| Gasperín-Rodríguez et al. (2022) | Veracruz | Clustering | *p*-value |
| Gónzalez-Bandala et al. (2020) | National | MLP | *r*, RMSE, RMSPE, MAPE |
| Gonzalez-Briceno et al. (2020) | Jalisco | CNN | Accuracy, Loss, Recall, Specificity, AUC |
| Gutiérrez-Esparza et al. (2020) | CDMX | RF | Accuracy, Recall, Specificity |
| Gutiérrez-Esparza et al. (2021) | CDMX | RF, C4.5, ANN | PPV, NPV, Accuracy, Balanced Accuracy |
| Guzmán-Torres et al. (2021) | National | Logistic Regression | Accuracy, Precision, Recall, F1-score, AUC |
| Martínez-Velasco et al. (2019) | National | RF | AUC, Accuracy, Average Accuracy, Precision, Recall, Specificity, F1-score |
| Morgan-Benita et al. (2022) | National | GLM, SVM, ANN | AUC, Recall, Specificity, Precision, Accuracy, F1-score, FPR, FNR, NPV |
| Muhammad et al. (2021) | National | Logistic Regression, Decision Tree, SVM, Naïve Bayes, ANN | Accuracy, Recall, Specificity |
| Pradhan et al. (2022) | National | Logistic Regresion, RF, XGBoost, AdaBoost, KNN, ANN | Accuracy, Precision, Recall, F1-score, AUC, Confusion Matrix |
| Prieto (2022) | National | Logistic Regresion, Decision Tree, KNN, Naïve Bayes, XGBoost, RF | Confusion Matrix |
| Quiroz-Juárez et al. (2021) | National | ANN, Logistic Regression, SVM, KNN | Accuracy, Recall, Sensitivity |
| Rojas-García et al. (2023) | National | SVM, XGBoost, RF, Logistic Regression | F1-score, Accuracy, Recall, Specificity, PPV, NPV |
| Rojas-Mendizabal et al. (2021) | Baja California | SVM, KNN, RF, Logistic Regression, Classification Tree | Accuracy, F1-score, Precision, Recall |
| Soto-Murillo et al. (2021) | National | KNN, Naïve Bayes, Decision Tree, Logistic Regression, SVM, ANN | Accuracy, Recall, Specificity, Precision, F1-score, AUC |
| Urrutia and Villalobos (2022) | National | C5.0 | AUC, Accuracy, Recall and Specificity |
| Vázquez et al. (2020) | National | KNN, ANN, RF | AUC |
| Geosciences | | | |
| Titos et al. (2020) | Colima | CNN, MLP, RF, SVM | Accuracy |
| Trejo-Alonso et al. (2021) | Querétaro | ANN | RMSE, R^2^, MAE |
| Bustillos et al. (2021) | CDMX | ANN | Accuracy, Precision, Recall, F1-score, among others |
| Social Sciences | | | |
| Ávila-Solís et al. (2022) | National | Logistic Regression, SVM, RF | Accuracy, F1-score, Precision, Recall, F-macro, AUC, P-S curve |
| Barreda-Luna et al. (2022) | Queretaro | ANN | RMSE, R^2^, MBE |
| Bello-Valle et al. (2022) | National | C4.5, CART, Naïve Bayes, SVC, KNN, AdaBoost, RF | Accuracy, F1-score |
| Castorena et al. (2021) | National | MLP | AUC, Recall, Specificity |
| Contreras-Hernández et al. (2023) | National | BERT, SVM, Naïve Bayes, Logistic Regression, Decision Tree | Precision, F1-score, Recall |
| Corona (2022) | National | RF | Accuracy, Precision, Recall, F1-score, R^2^ |
| González-Rossano et al. (2023) | National | Linear Regression, RF, PCA | MAE |
| Gutiérrez et al. (2018) | Aguascalientes | RF, SVM | Accuracy, Recall, Specificity, AUC |
| Gutiérrez-Esparza et al. (2019) | National | RF | Accuracy, Recall, Specificity, Kappa |
| Orozco-Ramírez et al. (2022) | Oaxaca | RF | Error Rate |
| Rodriguez‐Barrios et al. (2021) | National | Bayesian Network | Precision |
| Salas-Rueda and Castañeda-Martínez (2021) | CDMX | Linear Regression | MSE |
| Salas-Rueda and Ramírez-Ortega (2021) | CDMX | Linear Regression | MSE |
| Salas-Rueda (2020) | CDMX | Linear Regression, Decision Tree | MSE |
| Salas-Rueda et al. (2020a) | CDMX | Linear Regression, Decision, Tree | MSE |
| Salas-Rueda et al. (2020b) | CDMX | Linear Regression, Decision Tree | MSE |
| Salas-Rueda et al. (2021a) | CDMX | Linear Regression | MSE |
| Salas-Rueda et al. (2021e) | CDMX | Linear Regression, Decision Tree | MSE |
| Salas-Rueda et al. (2021b) | CDMX | Linear Regression | MSE |
| Salas-Rueda et al. (2021d) | CDMX | Linear Regression, Decision Tree | MSE |
| Salas-Rueda et al. (2022c) | CDMX | Linear Regression, Decision Tree | MSE |
| Salas-Rueda et al. (2021c) | CDMX | Linear Regression, Decision Tree | MSE |
| Salas-Rueda et al. (2022a) | CDMX | Linear Regression, Decision Tree | MSE |
| Salas-Rueda et al. (2022b) | CDMX | Linear Regression, Decision Tree | MSE |
| Salas-Rueda et al. (2022d) | CDMX | Linear Regression, Decision Tree | MSE |
| Saldana-Perez et al. (2019) | CDMX | SVM | Accuracy |
| Terán-Bustamante et al. (2021) | CDMX | SVM, Naïve Bayes, ANN | AUC, F1-score, Precision, Recall |
| Urbina-Nájera and Hernández-Calva (2022) | CDMX | Clustering | N/A |
| Rincón (2023) | National | Logistic Regression, RF, SVM | Accuracy, Recall, Specificity, Precision, NPV, F1-score, Kappa |
| Agricultural and biological sciences | | | |
| Barreras et al. (2023) | National | RF, SVM, ANN, GLM | R^2^, RMSE |
| Campos-Ferreira and González-Camacho (2021) | Morelos | CNN | Accuracy, Recall, Precision, F1_s |
| Gómez et al. (2021b) | National | SVM, RF, GLM | R^2^, %RMSE |
| Gómez et al. (2021a) | National | GLM, Ridge regression, Partial Least Square Regression, KNN, SVM, XGBoost, RF | R^2^, RMSE |
| Gomez-Flores et al. (2022) | National | CNN, MLP | Accuracy, Recall, Specificity, Mattews Correlation Coefficient (MCC) |
| Hernández-Moreno et al. (2021) | Puebla | RF | Accuracy, Kappa |
| Huang et al. (2022) | National | PCA, Partial Least Square Regression | N/A |
| Olguín-Rojas et al. (2022) | National | CNN | Accuracy, Recall, F1-score, Precision |
| Romero-Sanchez et al. (2022) | CDMX, Oaxaca, Michoacán | RF, GLM | Accuracy, AUC, Recall, Specificity |
| Servín-Palestina et al. (2022) | Zacatecas | MLP | R^2^, MAE, RMSE, MSE, NSE |
| Zamora-Gutierrez et al. (2016) | National | RF | Accuracy |
| Environmental Sciences | | | |
| Avila-Perez et al. (2023) | Guerrero | Logistic Regression, SVM | Accuracy, Precision, Recall |
| Cruz-López et al. (2019) | National | RF | RMSE |
| Fernández del Castillo et al. (2022) | Jalisco | Linear Regression, MLR, Logistic Regression | RSE, MSE, R^2^ |
| Gao et al. (2023) | Jalisco | ANN, RF, Logistic Regression | Accuracy |
| Hartman et al. (2022) | Michoacán | SVM | Accuracy |
| Illoldi-Rangel et al. (2008) | Oaxaca | GA | N/A |
| Kampichler et al. (2010) | Yucatán | RF, Classification Trees | Accuracy |
| Knappett et al. (2020) | Guanajuato | PCA, RF | R^2^, RMSE |
| Maciel-Nájera et al. (2021) | Chihuahua | RF | AUC, Recall, Specificity |
| Powlen et al. (2023) | National | RF | R^2^, MAE |
| Prieto-Amparán et al. (2019) | Chihuahua | SVM | Fuzzy Similarity Index |
| Quintero et al. (2014) | National | RF | Precision, F1-score, Recall |
| Ramos-Bernal et al. (2021) | Guerrero | KNN, SVM, AdaBoost | Accuracy, Precision, Recall, F1-score, Kappa |
| Tello-Mijares and Flores (2016) | Coahuila, Durango | RF, MLP, Bayesian Network | Sensitivity, Precision, FPR, Harmonic Mean |
| Torres-Vera (2023) | Guadalajara | MLR | R^2^, RMSE |
| Economics, Econometrics and Finance | | | |
| Cuaya-Simbro et al. (2022) | CDMX | CNN | Accuracy, Confusion Matrix |
| Dobler-Morales et al. (2022) | Oaxaca | RF, Cluster | Error rate, Confusion Matrix |
| Rocha-Salazar et al. (2021) | National | ANN | Accuracy, Error rate |
| Energy | | | |
| Cruz-May et al. (2021) | Yucatán | ANFIS, ANN, GMDH | RMSE, R^2^ |
| Ibargüengoytia-González et al. (2021) | Oaxaca | Bayesian Network | Error rate |
| Ramirez-Gonzalez et al. (2022) | Baja California Sur | CNN, SVM, KNN | Accuracy |
|  |  |  |  |
| Villegas-Mier et al. (2022) | Queretaro | RF, Adaboost, LR, RNN, SVM | Accuracy, MSE, RMSE, MAE, MAPE, R^2^ |
| Arts and Humanities | | | |
| López-García et al. (2020) | Tlaxcala | PCA, Cluster | -- |
| Ramírez-Eudave et al. (2023) | Morelos | RF | Accuracy |
| Neurosciences | | | |
| Delgado-Gallegos et al. (2023) | National | C5.0 | Accuracy |
| Gomez-Cravioto et al. (2021) | National | RNN | RMSE |
| Mathematics | | | |
| Pérez-Ortega et al. (2022) | National | Cluster | -- |
| Biochemistry | | | |
| Sánchez-Delacruz et al. (2019) | Tabasco | MLP, AdaBoost | Accuracy |
| Business, Management and Accounting | | | |
| López-Chau et al. (2022) | Hidalgo | ANN | RMSE, MAE, R^2^ |
| Political Sciences | | | |
| Cantú (2019) | National | CNN | Accuracy |

Table S1. Extracted information of the 120 reviewed articles. Graphical information can be found across Section 3.

**References**

Ahmad, M., Rappenglück, B., Osibanjo, O. O., and Retama, A. (2022). A machine learning approach to investigate the build-up of surface ozone in Mexico-City. *J Clean Prod* 379. doi: 10.1016/j.jclepro.2022.134638

Almustafa, K. M. (2021). Covid19-Mexican-Patients’ Dataset (Covid19MPD) Classification and Prediction Using Feature Importance. *Concurr Comput* 34. doi: 10.1002/cpe.6675

Appice, A., Gel, Y. R., Iliev, I., Lyubchich, V., and Malerba, D. (2020). A Multi-Stage Machine Learning Approach to Predict Dengue Incidence: A Case Study in Mexico. *IEEE Access* 8, 52713–52725. doi: 10.1109/ACCESS.2020.2980634

Arellano-Verdejo, J., and Lazcano-Hernández, H. E. (2021). Collective view: Mapping Sargassum distribution along beaches. *PeerJ Comput Sci* 7. doi: 10.7717/peerj-cs.528

Arellano-Verdejo, J., Lazcano-Hernandez, H. E., and Cabanillas-Terán, N. (2019). ERISNet: Deep neural network for sargassum detection along the coastline of the mexican caribbean. *PeerJ* 2019. doi: 10.7717/peerj.6842

Argueta-Santillan, M., Campos-Castolo, E. M., Méndez-Lucero, M. Á., Lima-Sánchez, D. N., Urbina-González, J. F., Cerón-Solís, O., et al. (2021). Use of artificial intelligence to evaluate the detection of retinal alterations as a screening test in Mexican patients. *International Journal of Combinatorial Optimization Problems and Informatics* 12, 79–86. Available at: www.editada.org

Arias-Rodriguez, L. F., Duan, Z., Sepúlveda, R., Martinez-Martinez, S. I., and Disse, M. (2020). Monitoring water quality of Valle de Bravo reservoir, Mexico, using entire lifespan of meris data and machine learning approaches. *Remote Sens (Basel)* 12. doi: 10.3390/rs12101586

Avila-Perez, H., Flores-Munguía, E. J., Rosas-Acevedo, J. L., Gallardo-Bernal, I., and Ramirez-delReal, T. A. (2023). Comparative Analysis of Water Quality Applying Statistic and Machine Learning Method: A Case Study in Coyuca Lagoon and Tecpan River, Mexico. *Water (Switzerland)* 15. doi: 10.3390/w15040640

Ávila-Solís, P. R., González-Camacho, J. M., Vargas-Chanes, D., and Peréz-Rodríguez, P. (2022). Prediction of social lag in Mexico: a Machine Learning approach from economic unit data. *Agrociencia* 56, 233–247. doi: 10.47163/agrociencia.v56i2.2768

Baak-Baak, C. M., Cigarroa-Toledo, N., Pinto-Castillo, J. F., Cetina-Trejo, R. C., Torres-Chable, O., Blitvich, B. J., et al. (2022). Cluster Analysis of Dengue Morbidity and Mortality in Mexico from 2007 to 2020: Implications for the Probable Case Definition. *American Journal of Tropical Medicine and Hygiene* 106, 1515–1521. doi: 10.4269/ajtmh.21-0409

Barreda-Luna, A. A., Rodríguez-Reséndiz, J., Rodríguez-Abreo, O., and Álvarez-Alvarado, J. M. (2022). Spatial Models and Neural Network for Identifying Sustainable Transportation Projects with Study Case in Querétaro, an Intermediate Mexican City. *Sustainability (Switzerland)* 14. doi: 10.3390/su14137796

Barreras, A., Alanís de la Rosa, J. A., Mayorga, R., Cuenca, R., Moreno-G, C., Godínez, C., et al. (2023). Spatial predictions of tree density and tree height across Mexico forests using ensemble learning and forest inventory data. *Ecol Evol* 13. doi: 10.1002/ece3.10090

Becerra-Rico, J., Aceves-Fernández, M. A., Esquivel-Escalante, K., and Pedraza-Ortega, C. J. (2020). Airborne particle pollution predictive model using Gated Recurrent Unit (GRU) deep neural networks. *Earth Sci Inform* 12, 821–834. doi: 10.1007/s12145-020-00462-9

Becerra-Sánchez, A., Rodarte-Rodríguez, A., Escalante-García, N. I., Olvera-González, J. E., de la Rosa-Vargas, J. I., Zepeda-Valles, G., et al. (2022). Mortality Analysis of Patients with COVID-19 in Mexico Based on Risk Factors Applying Machine Learning Techniques. *Diagnostics* 12. doi: 10.3390/diagnostics12061396

Bello-Valle, A. S., Martínez-Rebollar, A., Sánchez, W., and Estrada-Esquivel, H. (2022). A Predictive Model for Automatic Detection of Loneliness and Social Isolation using Machine Learning. *Computacion y Sistemas* 26, 113–124. doi: 10.13053/CyS-26-1-4157

Bustillos, A., Santiago, C., and Rubín, G. (2021). Earthquakes Insights and Predictions in Mexico Using Machine Learning. *Research in Computing Science* 150, 7–20.

Campos-Ferreira, U. E., and González-Camacho, J. M. (2021). Convolutional Neural Network  classifier for identifying diseases of avocato fruit (Persea americana Mill.) from digital images. *Agrociencia* 55, 695–709. doi: 10.47163/agrociencia.v55i8.2662

Cantú, F. (2019). The fingerprints of fraud: Evidence from Mexico’s 1988 presidential election. *American Political Science Review* 113, 710–726. doi: 10.1017/S0003055419000285

Carmona, J. M., Gupta, P., Lozano-García, D. F., Vanoye, A. Y., Hernández-Paniagua, I. Y., and Mendoza, A. (2021). Evaluation of modis aerosol optical depth and surface data using an ensemble modeling approach to assess pm2.5 temporal and spatial distributions. *Remote Sens (Basel)* 13. doi: 10.3390/rs13163102

Carrillo-Vega, M. F., Pérez-Zepeda, M. U., Salinas-Escudero, G., García-Peña, C., Reyes-Ramírez, E. D., Espinel-Bermúdez, M. C., et al. (2022). Patterns of Muscle-Related Risk Factors for Sarcopenia in Older Mexican Women. *Int J Environ Res Public Health* 19. doi: 10.3390/ijerph191610239

Castillo-Olea, C., Conte-Galván, R., Zuñiga, C., Siono, A., Huerta, A., Bardhi, O., et al. (2021). Early stage identification of COVID-19 patients in mexico using machine learning: A case study for the Tijuana general hospital. *Information (Switzerland)* 12. doi: 10.3390/info12120490

Castillo-Olea, C., Soto, B. G. Z., Carballo-Lozano, C., and Zuñiga, C. (2019). Automatic classification of sarcopenia level in older adults: A case study at Tijuana General Hospital. *Int J Environ Res Public Health* 16. doi: 10.3390/ijerph16183275

Castillo-Olea, C., Soto, B. G. Z., and Zuñiga, C. (2020). Evaluation of prevalence of the sarcopenia level using machine learning techniques: Case study in tijuana baja california, mexico. *Int J Environ Res Public Health* 17. doi: 10.3390/ijerph17061917

Castorena, C. M., Abundez, I. M., Alejo, R., Granda-Gutiérrez, E. E., Rendón, E., and Villegas, O. (2021). Deep Neural Network for gender-based violence detection on twitter messages. *Mathematics* 9. doi: 10.3390/math9080807

Chadaga, K., Prabhu, S., Umakanth, S., Bhat, V. K., Sampathila, N., Chadaga, R. P., et al. (2021). COVID-19 Mortality Prediction among Patients Using Epidemiological Parameters: An Ensemble Machine Learning Approach. *Engineered Science* 16, 221–233. doi: 10.30919/es8d579

Contreras-Hernández, S., Tzili-Cruz, M. P., Espínola-Sánchez, J. M., and Pérez-Tzili, A. (2023). Deep Learning Model for COVID-19 Sentiment Analysis on Twitter. *New Gener Comput* 41, 189–212. doi: 10.1007/s00354-023-00209-2

Contreras-Navarro, E., García-Cueto, O. R., González-Navarro, F. F., and Valenzuela-Palacios, E. A. (2016). Modelado de las temperaturas del aire a 850 milibares: Un potencial indicador de las ondas cálidas en el noroeste de México. *Informacion Tecnológica* 27, 141–152. doi: 10.4067/S0718-07642016000200017

Coria, S. R., Gay-García, C., Villers-Ruiz, L., Guzmán-Arenas, A., Sánchez-Meneses, O., Ávila-Barrón, O. R., et al. (2016). Climate patterns of political division units obtained using automatic classification trees. *Atmósfera* 29, 359–377. doi: 10.20937/ATM.2016.29.04.06

Corona, A. (2022). Crisis in Mexico: the effect of the president’s discourse on state-level government communication about Covid-19 on Twitter. *Media e Jornalismo* 22, 199–218. doi: 10.14195/2183-5462_40_10

Cruz-López, M. I., Manzo-Delgado, L. de L., Aguirre-Gómez, R., Chuvieco, E., and Equihua-Benítez, J. A. (2019). Spatial distribution of forest fire emissions: A case study in three Mexican ecoregions. *Remote Sens (Basel)* 11. doi: 10.3390/rs11101185

Cruz-May, E., Bassam, A., Ricalde, L. J., Escalante-Soberanis, M. A., Oubram, O., May Tzuc, O., et al. (2021). Global sensitivity analysis for a real-time electricity market forecast by a machine learning approach: A case study of Mexico. *International Journal of Electrical Power and Energy Systems* 135. doi: 10.1016/j.ijepes.2021.107505

Cuaya-Simbro, G., Hernández-Vera, I., Ruiz, E., and Gutiérrez-Fragoso, K. (2022). Automatic Tariff Classification System using Deep Learning. *International Journal of Advanced Computer Science and Applications* 13, 904–911. doi: 10.14569/issn.2156-5570

Delgado-Gallegos, J. L., Avilés-Rodriguez, G., Padilla-Rivas, G. R., De los Ángeles Cosío-León, M., Franco-Villareal, H., Nieto-Hipólito, J. I., et al. (2023). Application of C5.0 Algorithm for the Assessment of Perceived Stress in Healthcare Professionals Attending COVID-19. *Brain Sci* 13. doi: 10.3390/brainsci13030513

Dobler-Morales, C., Lorenzen, M., Orozco-Ramírez, Q., and Bocco, G. (2022). Beyond a generalized deagrarianization: Livelihood heterogeneity and its determinants in the Mixteca Alta, Mexico. *World Dev* 160. doi: 10.1016/j.worlddev.2022.106074

Espinosa-Guzmán, A. A., May-Tzuc, O., Balam-Pantí, I., Reyes-Trujeque, J., Pérez-Quintana, I. V., and Bassam, A. (2017). Modelado de partículas PM10 y PM2.5 mediante redes neuronales artificiales sobre clima tropical de San Francisco de Campeche, México. *Quim Nova* 40, 1025–1034. doi: 10.21577/0100-4042.20170115

Fernández del Castillo, A., Yebra-Montes, C., Garibay, M. V., de Anda, J., Garcia-Gonzalez, A., and Gradilla-Hernández, M. S. (2022). Simple Prediction of an Ecosystem-Specific Water Quality Index and the Water Quality Classification of a Highly Polluted River through Supervised Machine Learning. *Water (Switzerland)* 14. doi: 10.3390/w14081235

Gallardo-Rincón, H., Ríos-Blancas, M. J., Ortega-Montiel, J., Montoya, A., Martinez-Juarez, L. A., Lomelín-Gascón, J., et al. (2023). MIDO GDM: an innovative artificial intelligence-based prediction model for the development of gestational diabetes in Mexican women. *Sci Rep* 13. doi: 10.1038/s41598-023-34126-7

Gao, Y., Solórzano, J. V., Estoque, R. C., and Tsuyuzaki, S. (2023). Tropical Dry Forest Dynamics Explained by Topographic and Anthropogenic Factors: A Case Study in Mexico. *Remote Sens (Basel)* 15. doi: 10.3390/rs15051471

Gasperín-Rodríguez, E. I., Gómez-Figueroa, J. A., Gómez-Miranda, L. M., Ríos-Gallardo, P. T., Palmeros-Exsome, C., Hernández-Lepe, M. A., et al. (2022). Body Composition Profiles of Applicants to a Physical Education and Sports Major in Southeastern Mexico. *Int J Environ Res Public Health* 19. doi: 10.3390/ijerph192315685

Gómez, D., Salvador, P., Sanz, J., and Casanova, J. L. (2021a). Modelling wheat yield with antecedent information, satellite and climate data using machine learning methods in Mexico. *Agric For Meteorol* 300. doi: 10.1016/j.agrformet.2020.108317

Gómez, D., Salvador, P., Sanz, J., and Casanova, J. L. (2021b). Regional estimation of garlic yield using crop, satellite and climate data in Mexico. *Comput Electron Agric* 181. doi: 10.1016/j.compag.2020.105943

Gomez-Cravioto, D. A., Diaz-Ramos, R. E., Cantu-Ortiz, F. J., and Ceballos, H. G. (2021). Data Analysis and Forecasting of the COVID-19 Spread: A Comparison of Recurrent Neural Networks and Time Series Models. *Cognit Comput* 2021. doi: 10.1007/s12559-021-09885-y

Gomez-Flores, W., Garza-Saldana, J. J., and Varela-Fuentes, S. E. (2022). A Huanglongbing Detection Method for Orange Trees Based on Deep Neural Networks and Transfer Learning. *IEEE Access* 10, 116686–116696. doi: 10.1109/ACCESS.2022.3219481

Gónzalez-Bandala, D. A., Cuevas-Tello, J. C., Noyola, D. E., Comas-García, A., and García-Sepúlveda, C. A. (2020). Computational forecasting methodology for acute respiratory infectious disease dynamics. *Int J Environ Res Public Health* 17, 1–20. doi: 10.3390/ijerph17124540

Gonzalez-Briceno, G., Sanchez, A., Ortega-Cisneros, S., Garcia-Contreras, M. S., Pinedo-Diaz, G. A., and Moya-Sanchez, E. U. (2020). Artificial Intelligence-Based Referral System for Patients with Diabetic Retinopathy. *Computer (Long Beach Calif)* 53, 77–87. doi: 10.1109/MC.2020.3004392

González-Rossano, C., Terán-Bustamante, A., Velázquez-Salazar, M., and Martínez-Velasco, A. (2023). What Drives Profit Income in Mexico’s Main Banks? Evidence Using Machine Learning. *Sustainability (Switzerland)* 15. doi: 10.3390/su15075696

Gutiérrez, G., Canul-Reich, J., Ochoa-Zezzatti, A., Margain, L., and Ponce, J. (2018). Mining: Students Comments about Teacher Performance Assessment using Machine Learning Algorithms. *International Journal of Combinatorial Optimization Problems and Informatics* 9, 26–40. Available at: www.market.android.com

Gutiérrez-Avila, I., Arfer, K. B., Carrión, D., Rush, J., Kloog, I., Naeger, A. R., et al. (2022). Prediction of daily mean and one-hour maximum PM2.5 concentrations and applications in Central Mexico using satellite-based machine-learning models. *J Expo Sci Environ Epidemiol* 32, 917–925. doi: 10.1038/s41370-022-00471-4

Gutiérrez-Esparza, G. O., Ramírez-Delreal, T. A., Martínez-García, M., Infante Vázquez, O., Vallejo, M., and Hernández-Torruco, J. (2021). Machine and deep learning applied to predict metabolic syndrome without a blood screening. *Applied Sciences (Switzerland)* 11. doi: 10.3390/app11104334

Gutiérrez-Esparza, G. O., Vallejo-Allende, M., and Hernández-Torruco, J. (2019). Classification of cyber-aggression cases applying machine learning. *Applied Sciences (Switzerland)* 9. doi: 10.3390/app9091828

Gutiérrez-Esparza, G. O., Vázquez, O. I., Vallejo, M., and Hernández-Torruco, J. (2020). Prediction of metabolic syndrome in a Mexican population applying machine learning algorithms. *Symmetry (Basel)* 12. doi: 10.3390/SYM12040581

Guzmán-Torres, J. A., Alonso-Guzmán, E. M., Domínguez-Mota, F. J., and Tinoco-Guerrero, G. (2021). Estimation of the main conditions in (SARS-CoV-2) Covid-19 patients that increase the risk of death using Machine learning, the case of Mexico. *Results Phys* 27. doi: 10.1016/j.rinp.2021.104483

Hartman, S., Farfán, M., Hoogesteger, J., and D’Odorico, P. (2022). Mapping the expansion of berry greenhouses onto Michoacán’s ejido lands, México. *Environmental Research Letters* 17. doi: 10.1088/1748-9326/ac9ac8

Hernández-Moreno, M. M., Téllez-Valdés, O., Martínez-Meyer, E., Islas-Saldaña, L. A., Salazar-Rojas, V. M., and Macías-Cuéllar, H. (2021). Distribución de la cobertura vegetal y del uso del terreno del municipio de Zapotitlán, Puebla, México. *Rev Mex Biodivers* 92. doi: 10.22201/ib.20078706e.2021.92.3649

Huang, J., Alanís-Martínez, I., Kumagai, L., Dai, Z., Zheng, Z., Perez de Leon, A. A., et al. (2022). Machine learning and analysis of genomic diversity of “Candidatus Liberibacter asiaticus” strains from 20 citrus production states in Mexico. *Front Plant Sci* 13. doi: 10.3389/fpls.2022.1052680

Ibargüengoytia-González, P. H., Reyes-Ballesteros, A., Borunda-Pacheco, M., and García López, U. A. (2021). Prediction of Wind Power Generation with Modern Artificial Intelligence Technology. *Journal of Autonomous Intelligence* 4, 52–62. doi: 10.32629/jai.v4i2.501

Illoldi-Rangel, P., Fuller, T., Linaje, M., Pappas, C., Sánchez-Cordero, V., and Sarkar, S. (2008). Solving the maximum representation problem to prioritize areas for the conservation of terrestrial mammals at risk in Oaxaca. *Divers Distrib* 14, 493–508. doi: 10.1111/j.1472-4642.2007.00458.x

Kampichler, C., Calmé, S., Weissenberger, H., and Arriaga-Weiss, S. L. (2010). Indication of a species in an extinction vortex: The ocellated turkey on the Yucatan peninsula, Mexico. *Acta Oecologica* 36, 561–568. doi: 10.1016/j.actao.2010.08.004

Knappett, P. S. K., Li, Y., Loza, I., Hernandez, H., Avilés, M., Haaf, D., et al. (2020). Rising arsenic concentrations from dewatering a geothermally influenced aquifer in central Mexico. *Water Res* 185. doi: 10.1016/j.watres.2020.116257

López-Chau, A., Muñoz-Chávez, J. P., and Valle-Cruz, D. (2022). Percepción de la calidad en restaurantes: un análisis mixto con redes neuronales. *Estudios Gerenciales* 38, 449–463. doi: 10.18046/j.estger.2022.165.5235

López-García, P. A., Argote, D. L., and Thrun, M. C. (2020). Projection-Based Classification of Chemical Groups for Provenance Analysis of Archaeological Materials. *IEEE Access* 8, 152439–152451. doi: 10.1109/ACCESS.2020.3016244

López-Serrano, P. M., Domínguez, J. L. C., Corral-Rivas, J. J., Jiménez, E., López-Sánchez, C. A., and Vega-Nieva, D. J. (2020). Modeling of aboveground biomass with landsat 8 oli and machine learning in temperate forests. *Forests* 11. doi: 10.3390/f11010011

Maciel-Nájera, J. F., González-Elizondo, M. S., Hernández-Díaz, J. C., López-Sánchez, C. A., Bailón-Soto, C. E., Carrillo-Parra, A., et al. (2021). Influence of environmental factors on forest understorey species in Northern Mexico. *Forests* 12. doi: 10.3390/f12091198

Magallanes-Quintanar, R., Galván-Tejada, C. E., Galván-Tejada, J. I., Méndez-Gallegos, S., Blanco-Macías, F., and Valdez-Cepeda, R. D. (2023). Artificial neural network models for prediction of standardized precipitation index in central Mexico. *Agrociencia* 57. doi: 10.47163/agrociencia.v57i1.2655

Martínez-Velasco, A., Martínez-Villaseñor, L., Miralles-Pechuán, L., Perez-Ortiz, A. C., Zenteno, J. C., and Estrada-Mena, F. J. (2019). The relevance of cataract as a risk factor for age-related macular degeneration: A machine learning approach. *Applied Sciences (Switzerland)* 9. doi: 10.3390/app9245550

Mejia-Zuluaga, P. A., Dozal, L., and Valdiviezo-N., J. C. (2022). Genetic Programming Approach for the Detection of Mistletoe Based on UAV Multispectral Imagery in the Conservation Area of Mexico City. *Remote Sens (Basel)* 14. doi: 10.3390/rs14030801

Morgan-Benita, J. A., Galván-Tejada, C. E., Cruz, M., Galván-Tejada, J. I., Gamboa-Rosales, H., Arceo-Olague, J. G., et al. (2022). Hard Voting Ensemble Approach for the Detection of Type 2 Diabetes in Mexican Population with Non-Glucose Related Features. *Healthcare (Switzerland)* 10. doi: 10.3390/healthcare10081362

Muhammad, L. J., Algehyne, E. A., Usman, S. S., Ahmad, A., Chakraborty, C., and Mohammed, I. A. (2021). Supervised Machine Learning Models for Prediction of COVID-19 Infection using Epidemiology Dataset. *SN Comput Sci* 2. doi: 10.1007/s42979-020-00394-7

Olguín-Rojas, J. C., Vasquez-Gomez, J. I., López-Canteñs, G. de J., and Herrera-Lozada, J. C. (2022). Clasificación de manzanas con Redes Neuronales Convolucionales. *Revista Fitotecnia Mexicana* 45, 369–378. doi: 10.35196/rfm.2022.3.369

Orozco-Ramírez, Q., Lorenzen, M., Fernández de Castro Martínez, G., and Cruz-Ramírez, M. A. (2022). Social and biophysical factors of the forest transition in the Mixteca Alta UNESCO Global Geopark. *Investigaciones Geograficas*. doi: 10.14350/rig.60465

Pech-May, F., Aquino-Santos, R., Rios-Toledo, G., and Posadas-Durán, J. P. F. (2022). Mapping of Land Cover with Optical Images, Supervised Algorithms, and Google Earth Engine. *Sensors* 22. doi: 10.3390/s22134729

Pérez-Ortega, J., Almanza-Ortega, N. N., Torres-Poveda, K., Martínez-González, G., Zavala-Díaz, J. C., and Pazos-Rangel, R. (2022). Application of Data Science for Cluster Analysis of COVID-19 Mortality According to Sociodemographic Factors at Municipal Level in Mexico. *Mathematics* 10. doi: 10.3390/math10132167

Powlen, K. A., Salerno, J., Jones, K. W., and Gavin, M. C. (2023). Identifying socioeconomic and biophysical factors driving forest loss in protected areas. *Conservation Biology* 37. doi: 10.1111/cobi.14058

Pradhan, A., Prabhu, S., Chadaga, K., Sengupta, S., and Nath, G. (2022). Supervised Learning Models for the Preliminary Detection of COVID-19 in Patients Using Demographic and Epidemiological Parameters. *Information (Switzerland)* 13. doi: 10.3390/info13070330

Prieto, K. (2022). Current forecast of COVID-19 in Mexico: A Bayesian and machine learning approaches. *PLoS One* 17. doi: 10.1371/journal.pone.0259958

Prieto-Amparán, J. A., Villarreal-Guerrero, F., Martínez-Salvador, M., Manjarrez-Domínguez, C., Vázquez-Quintero, G., and Pinedo-Alvarez, A. (2019). Spatial near future modeling of land use and land cover changes in the temperate forests of Mexico. *PeerJ* 2019. doi: 10.7717/peerj.6617

Quej, V. H., De La Cruz Castillo, C., Almorox, J., and Rivera-Hernandez, B. (2022). Evaluation of artificial intelligence models for daily prediction of reference evapotranspiration using temperature, rainfall and relative humidity in a warm sub-humid environment. *Italian Journal of Agrometeorology* 2022, 49–63. doi: 10.36253/ijam-1373

Quintero, E., Thessen, A. E., Arias-Caballero, P., and Ayala-Orozco, B. (2014). A statistical assessment of population trends for data deficient Mexican amphibians. *PeerJ* 2014. doi: 10.7717/peerj.703

Quiroz-Juárez, M. A., Torres-Gómez, A., Hoyo-Ulloa, I., de León-Montiel, R. D. J., and U’Ren, A. B. (2021). Identification of high-risk COVID-19 patients using machine learning. *PLoS One* 16. doi: 10.1371/journal.pone.0257234

Ramírez-Eudave, R., Ferreira, T. M., Vicente, R., Lourenco, P. B., and Peña, F. (2023). Parametric and Machine Learning-Based Analysis of the Seismic Vulnerability of Adobe Historical Buildings Damaged After the September 2017 Mexico Earthquakes. *International Journal of Architectural Heritage*. doi: 10.1080/15583058.2023.2200739

Ramirez-Gonzalez, M., Segundo Sevilla, F. R., Korba, P., and Castellanos-Bustamante, R. (2022). Convolutional neural nets with hyperparameter optimization and feature importance for power system static security assessment. *Electric Power Systems Research* 211. doi: 10.1016/j.epsr.2022.108203

Ramos-Bernal, R. N., Vázquez-Jiménez, R., Cantú-Ramírez, C. A., Alarcón-Paredes, A., Alonso-Silverio, G. A., Bruzón, A. G., et al. (2021). Evaluation of conditioning factors of slope instability and continuous change maps in the generation of landslide inventory maps using machine learning (ML) algorithms. *Remote Sens (Basel)* 13. doi: 10.3390/rs13224515

Ramos-Cirilo, L. A., Quej-Chi, V. H., Carrillo-Ávila, E., Navarro, E. A., and Rivera-Hernández, B. (2021). Estimation of reference evapotranspiration from temperature data: A comparison between conventional calculation and artificial intelligence techniques in a warm-sub-humid region. *Tecnologia y Ciencias del Agua* 12, 1–33. doi: 10.24850/j-tyca-2021-03-02

Rincón, R. (2023). Estimaciones trimestrales de pobreza multidimensional en México mediante algoritmos de aprendizaje de máquina. *Estudios Económicos de El Colegio de México* 38, 3–68. doi: 10.24201/ee.v38i1.435

Rocha-Salazar, J. de J., Segovia-Vargas, M. J., and Camacho-Miñano, M. del M. (2021). Money laundering and terrorism financing detection using neural networks and an abnormality indicator. *Expert Syst Appl* 169. doi: 10.1016/j.eswa.2020.114470

Rodriguez‐Barrios, E. U., Melendez‐Armenta, R. A., Garcia‐Aburto, S. G., Lavoignet‐Ruiz, M., Sandoval‐Herazo, L. C., Molina‐Navarro, A., et al. (2021). Bayesian approach to analyze reading comprehension: A case study in elementary school children in Mexico. *Sustainability (Switzerland)* 13. doi: 10.3390/su13084285

Rojas-García, M., Vázquez, B., Torres-Poveda, K., and Madrid-Marina, V. (2023). Lethality risk markers by sex and age-group for COVID-19 in Mexico: a cross-sectional study based on machine learning approach. *BMC Infect Dis* 23. doi: 10.1186/s12879-022-07951-w

Rojas-Mendizabal, V., Castillo-Olea, C., Gómez-Siono, A., and Zuñiga, C. (2021). Assessment of thoracic pain using machine learning: A case study from Baja California, Mexico. *Int J Environ Res Public Health* 18, 1–12. doi: 10.3390/ijerph18042155

Romero-Sanchez, M. E., Velasco-Garcia, M. V., Perez-Miranda, R., Velasco-Bautista, E., and Gonzalez-Hernandez, A. (2022). Different Modelling Approaches to Determine Suitable Areas for Conserving Egg-Cone Pine (Pinus oocarpa Schiede) Plus Trees in the Central Part of Mexico. *Forests* 13. doi: 10.3390/f13122112

Salas-Rueda, R. A. (2020). Percepciones de los estudiantes sobre el uso de Facebook y Twitter en el contexto educativo por medio de la ciencia de datos y el aprendizaje automático. *Pixel-Bit, Revista de Medios y Educacion* 58, 91–115. doi: 10.12795/pixelbit.74056

Salas-Rueda, R. A., Alvarado-Zamorano, C., and Ramírez-Ortega, J. (2022a). Construction of a Web Game for the Teaching-Learning Process of Electronics during the COVID-19 Pandemic. *Educational Process: International Journal* 11, 130–146. doi: 10.22521/edupij.2022.112.7

Salas-Rueda, R. A., and Castañeda-Martínez, R. (2021). Opinión de docentes sobre los dispositivos móviles considerando la ciencia de datos. *Revista Fuentes*, 163–177. doi: 10.12795/revistafuentes.2021.12292

Salas-Rueda, R. A., Castañeda-Martínez, R., Eslava-Cervantes, A., and Alvarado-Zamorano, C. (2022b). Teachers’ Perception About MOOCs and ICT During the COVID-19 Pandemic. *Contemp Educ Technol* 14. doi: 10.30935/cedtech/11479

Salas-Rueda, R. A., Castañeda-Martínez, R., Ramírez-Ortega, J., and Alvarado -Zamorano, C. (2022c). Análisis sobre el uso de la tecnología en la asignatura Método Clínico durante la pandemia Covid-19 considerando la ciencia de datos. *Digital Education Review* 41, 195–223.

Salas-Rueda, R. A., Castañeda-Martínez, R., Ramírez-Ortega, J., and Gamboa-Rodríguez, F. (2020a). Análisis sobre el uso de Podcast en la Escuela Nacional de Trabajo Social considerando la ciencia de datos y el aprendizaje automático. *Revista de Gestión de las Personas y Tecnología* 37, 68–80. Available at: www.revistagpt.usach.cl

Salas-Rueda, R. A., Castañeda-Martínez, R., Ramírez-Ortega, J., and Garcés-Madrigal, A. M. (2021a). Opinión de los educadores sobre la tecnología y las plataformas web durante la pandemia Covid-19. *Revista Gestión de las Personas y Tecnología* 40.

Salas-Rueda, R. A., De-La-Cruz-Martínez, G., Castañeda-Martínez, R., and Alvarado-Zamorano, C. (2022d). Percepción de los estudiantes sobre el uso de las plataformas LMS y los teléfonos inteligentes durante la pandemia Covid-19. *Meta: Avaliacao* 14, 237–261. doi: 10.22347/2175-2753v14i43.3661

Salas-Rueda, R. A., Eslava-Cervantes, A. L., and Prieto-Larios, E. (2020b). Teachers’ perceptions about the impact of moodle in the educational field considering data science. *Online J Commun Media Technol* 10. doi: 10.30935/ojcmt/8498

Salas-Rueda, R. A., Eslava-Cervantes, A. L., and Prieto-Larios, E. (2021b). Analysis of the impact of flipped classroom and technology in the educational process on the Design of Graphic Communication. *Vivat Academia. Revista de Comunicación* 2021, 25–39. doi: 10.15178/va.2021.154.e1238

Salas-Rueda, R. A., and Ramírez-Ortega, J. (2021). Students’ perceptions about the use of flipped classroom in the field of electronic electrical engineering. *Br. J. Ed., Tech. Soc* 14, 158–166. doi: 10.14571/brajets.v14.n1

Salas-Rueda, R. A., Ramírez-Ortega, J., Eslava-Cervantes, A., Castañeda-Martínez, R., and De-La-Cruz-Martínez, G. (2021c). Percepción de los profesores sobre los juegos web y dispositivos móviles en el nivel educativo superior durante la pandemia COVID-19. *Texto Livre* 15. doi: 10.35699/1983-3652.2022.37074

Salas-Rueda, R. A., Ramírez-Ortega, J., and Eslava-Cervantes, A. L. (2021d). Use of the collaborative wall to improve the teaching-learning conditions in the bachelor of visual arts. *Contemp Educ Technol* 13, 1–10. doi: 10.30935/cedtech/8711

Salas-Rueda, R.-A., Jiménez-Bandala, C.-A., and Alvarado-Zamorano, C. (2021e). Schoology: a web platform capable of improving the teaching-learning process at the higher educational level. *Revista de Comunicación de la SEECI* 2021, 19–41. doi: 10.15198/seeci.2021.54.e645

Saldana-Perez, M., Torres-Ruiz, M., and Moreno-Ibarra, M. (2019). Geospatial Modeling of Road Traffic Using a Semi-Supervised Regression Algorithm. *IEEE Access* 7, 177376–177386. doi: 10.1109/ACCESS.2019.2942586

Salvador, P., Gómez, D., Sanz, J., and Casanova, J. L. (2020). Estimation of potato yield using satellite data at a municipal level: A machine learning approach. *ISPRS Int J Geoinf* 9. doi: 10.3390/ijgi9060343

Sánchez-Delacruz, E., Weber, R., Biswal, R. R., Mejía, J., Hernández-Chan, G., and Gómez-Pozos, H. (2019). Gait Biomarkers Classification by Combining Assembled Algorithms and Deep Learning: Results of a Local Study. *Comput Math Methods Med* 2019. doi: 10.1155/2019/3515268

Schulthess, U., Rodrigues, F., Taymans, M., Bellemans, N., Bontemps, S., Ortiz-Monasterio, I., et al. (2023). Optimal Sample Size and Composition for Crop Classification with Sen2-Agri’s Random Forest Classifier. *Remote Sens (Basel)* 15. doi: 10.3390/rs15030608

Servín-Palestina, M., Salazar-Moreno, R., López-Cruz, I., Medina-García, G., and Cid-Ríos, J. (2022). Predicción de la producción y rendimiento de frijol, con modelos de redes neuronales artificiales y datos climáticos Prediction of bean production and yields, with artificial neural network models and climate data. *Revista de Ciencias Biológicas y de la Salud* 24. Available at: http://biotecnia.unison.mx

Soto-Murillo, M. A., Galván-Tejada, J. I., Galván-Tejada, C. E., Celaya-Padilla, J. M., Luna-García, H., Magallanes-Quintanar, R., et al. (2021). Automatic evaluation of heart condition according to the sounds emitted and implementing six classification methods. *Healthcare (Switzerland)* 9. doi: 10.3390/healthcare9030317

Tello-Mijares, S., and Flores, F. (2016). A novel method for the separation of overlapping pollen species for automated detection and classification. *Comput Math Methods Med* 2016. doi: 10.1155/2016/5689346

Terán-Bustamante, A., Martínez-Velasco, A., and Dávila-Aragón, G. (2021). Knowledge Management for Open Innovation: Bayesian Networks through Machine Learning. *Journal of Open Innovation: Technology, Market, and Complexity Article* 7. doi: 10.3390/joitmc7010040

Titos, M., Bueno, A., García, L., Benítez, C., and Segura, J. C. (2020). Classification of Isolated Volcano-Seismic Events Based on Inductive Transfer Learning. *IEEE Geoscience and Remote Sensing Letters* 17, 869–873. doi: 10.1109/LGRS.2019.2931063

Torres-Vera, M. A. (2023). Mapping of total suspended solids using Landsat imagery and machine learning. *International Journal of Environmental Science and Technology* 20, 11877–11890. doi: 10.1007/s13762-023-04787-y

Trejo-Alonso, J., Fuentes, C., Chávez, C., Quevedo, A., Gutierrez-Lopez, A., and González-Correa, B. (2021). Saturated hydraulic conductivity estimation using artificial neural networks. *Water (Switzerland)* 13. doi: 10.3390/w13050705

Urbina-Nájera, A. B., and Hernández-Calva, J. C. (2022). Reducción del estrés en el personal de empresas de servicios tecnológicos durante la pandemia por COVID-19 aplicando clustering. *Revista San Gregorio* 2022, 36–57.

Urrutia, J. E. S., and Villalobos, M. A. (2022). An empirical analysis of homicides in Mexico through Machine Learning and statistical design of experiments. *Poblac Salud Mesoam* 20. doi: 10.15517/psm.v20i1.48217

Vázquez, A. L., Domenech Rodríguez, M. M., Barrett, T. S., Schwartz, S., Amador Buenabad, N. G., Bustos Gamiño, M. N., et al. (2020). Innovative Identification of Substance Use Predictors: Machine Learning in a National Sample of Mexican Children. *Prevention Science* 21, 171–181. doi: 10.1007/s11121-020-01089-4

Villegas-Mier, C. G., Rodriguez-Resendiz, J., Álvarez-Alvarado, J. M., Jiménez-Hernández, H., and Odry, Á. (2022). Optimized Random Forest for Solar Radiation Prediction Using Sunshine Hours. *Micromachines (Basel)* 13. doi: 10.3390/mi13091406

Zamora-Gutierrez, V., Lopez-Gonzalez, C., MacSwiney Gonzalez, M. C., Fenton, B., Jones, G., Kalko, E. K. V., et al. (2016). Acoustic identification of Mexican bats based on taxonomic and ecological constraints on call design. *Methods Ecol Evol* 7, 1082–1091. doi: 10.1111/2041-210X.12556

Zhu, L., and Aguilera, P. (2021). Evaluating Variations in Tropical Cyclone Precipitation in Eastern Mexico Using Machine Learning Techniques. *Journal of Geophysical Research: Atmospheres* 126. doi: 10.1029/2021JD034604
